# Controlled Triazine‐Based Covalent Functionalization of Black Phosphorus for Degradable Hybrid Materials

**DOI:** 10.1002/smll.73572

**Published:** 2026-04-27

**Authors:** Jasmin Er, Sreejita Ray, Enyu Xie, Robert Schusterbauer, Maik Rosentreter, Ranen Etouki, Na Xing, Anja Wiesner, Philip Nickl, Rameez Ahmed, Taylor Page, Ana Hočevar, Obida Bawadkji, Andreas Herrmann, Jörg Radnik, Vasile‐Dan Hodoroaba, Christian Sieben, Beate Paulus, Ievgen S. Donskyi

**Affiliations:** ^1^ Institut für Chemie und Biochemie Freie Universität Berlin Berlin Germany; ^2^ Federal Institute for Material Research and Testing (BAM), Division 6.1 Berlin Germany; ^3^ Institut für Chemie und Biochemie, Physikalische und Theoretische Chemie Freie Universität Berlin Berlin Germany; ^4^ Nanoscale Infection Biology Group Helmholtz Centre for Infection Research Braunschweig Germany

**Keywords:** advanced surface characterization, black phosphorus, bonding motifs, covalent functionalization, DFT calculations, virus inhibition

## Abstract

Controllable covalent surface functionalization of black phosphorus (BP) remains a central challenge in the development of 2D phosphorus‐based materials. Here, we report a scalable route to synthesize biodegradable BP‐polymer hybrids and establish optimal conditions for BP production, exfoliation, and covalent modification. BP sheets, produced via optimized mechanochemical and exfoliation processes, are covalently functionalized with 2‐azido‐4,6‐dichloro‐1,3,5‐triazine via a nitrene‐mediated [2+1] cycloaddition. The reaction yields a P‐N bond, verified by advanced surface analyses and density functional theory (DFT) calculations. The conjugated triazine groups enable subsequent nucleophilic aromatic substitution reactions, providing a versatile platform for controlled post‐modification of BP surface. This covalent functionalization strategy addresses key limitations in BP surface chemistry and provides a route toward biodegradable phosphorus‐based hybrid materials. As a representative example, functionalization with linear polyglycerol sulfate produces BP‐polymer conjugates that inhibit respiratory syncytial virus (RSV) and herpes simplex virus 1 (HSV‐1) at low‐microgram‐per‐milliliter concentrations.

## Graphical abstract

A scalable triazine‐based covalent functionalization strategy of black phosphorus nanosheets provides controlled P‐N surface chemistry with enhanced grafting density in the presence of a phase‐transfer catalyst. As a representative example, BP‐sulfated polymer conjugates exhibit strong in vitro antiviral activity against enveloped viruses and demonstrate the functional relevance of the controlled surface modification.

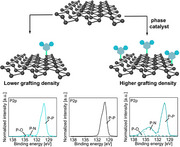

## 1 Introduction

BP has emerged as a distinct 2D material that degrades under ambient or aqueous conditions [1, 2, 3, 4]. During this process, physiologically compatible phosphate ions are produced that can be readily metabolized by the body [5, 6, 7]. In contrast to most 2D materials, the combination of BP's chemical properties, puckered lattice, and high specific surface area, make BP particularly attractive for diverse biomedical applications [8, 9, 10, 11, 12, 13]. To stabilize degradable BP and enable the formation of functional BP‐based materials, precise surface modification is essential [14]. Surface P atoms allow controlled functionalization with diverse moieties [14, 15, 16, 17]. For instance, treatment of exfoliated BP with organic azides forms stable P‐N bonds that significantly improve stability and suppress rapid oxidative degradation under ambient conditions [17]. Another well‐established route uses aryl diazonium salts, such diazonium‐induced arylation, which yields P─C bonds that suppress BP degradation for weeks under ambient conditions [15]. More recently, the functionalization strategies have expanded further. Nucleophilic reagents and isocyanates have been used to generate carbamate‐ or urethane‐type P─O─C and P─N─C bonds [18]. These examples highlight that BP surface chemistry supports multiple reaction pathways, beyond the arylation‐based strategies that dominated early studies [19]. Altogether, covalently attached groups stabilize the BP surface and create functional groups for further substitution [20]. However, existing covalent approaches often yield heterogeneous structures with low grafting densities [21]. This limits their suitability for biomedical applications that require uniform, well‐defined surface chemistry at functional interfaces. A surface functionalization strategy that establishes a structurally verified binding motif and enables controlled post‐modification would offer a significant advantage over existing approaches. Cyanuric chloride is a versatile linker that enables controlled functionalization at discrete temperatures [22]. Its three chlorine atoms undergo stepwise nucleophilic aromatic substitution in a temperature‐dependent manner, as the reactivity decreases after each substitution [22, 23]. Although related strategies have been reported for carbon‐based materials [24, 25], their direct application to BP remains unexplored.

Precise surface functionalization is particularly critical when BP is used in biological systems involving cell adhesion or pathogen interactions. These processes strongly depend on surface charge, functionalization density, and the distribution of functional groups [26, 27]. In particular, several enveloped viruses, including RSV and HSV‐1, rely on interactions with surface glycans on host cells, most prominently heparan sulfate proteoglycans, to initiate the infection [28, 29]. These interactions depend on electrostatic forces between viral surface proteins and negatively charged functional groups on the cell's glycocalyx [29, 30]. Because viral attachment is largely driven by complementary charges rather than virus‐specific receptors, anionic inhibitors can mimic and compete with heparan sulfate proteoglycan‐mediated recognition [28, 31]. Linear polymers functionalized with anionic sulfate or sulfonate groups, such as polystyrene sulfonate and sulfated polyvinyl alcohol, interfere with glycan‐mediated interactions [25, 28, 32]. This competition alters viral attachment without reliance on virus‐specific pathways [29, 33].

In this work, we present a new scalable covalent conjugation strategy for BP. The electrophilic nature of triazine moieties enables covalent P‐N bond formation under mild conditions, and the conjugated triazine groups provide two additional reactive sites for post‐functionalization. The controlled generation of a nitrene intermediate and the subsequent formation of a defined P‐N bond provides a platform establishes a platform for precise surface functionalization that has not been accessible with previous BP functionalization methods. The produced materials are characterized through a combination of advanced spectroscopic and microscopic techniques, including X‐ray photoelectron spectroscopy (XPS), near‐edge X‐ray absorption fine structure spectroscopy (NEXAFS), hard X‐ray photoelectron spectroscopy (HAXPES), Raman spectroscopy, X‐ray diffraction (XRD), atomic force microscopy (AFM), and time‐of‐flight secondary ion mass spectrometry (ToF‐SIMS). We quantify triazine grafting density and identify the dominant binding motif through DFT. Additionally, we demonstrate substitution of the conjugated surface moieties with linear polymer chains, yields functional BP‐based hybrids. Finally, BP‐polymer conjugates are evaluated in vitro and demonstrate inhibition of enveloped viruses such as HSV‐1 and RSV. Overall, this approach establishes a scalable covalent functionalization strategy for the synthesis of BP‐polymer hybrids and extends naturally to other covalently modified BP systems.

## 2 Results and Discussion

**FIGURE 1:**
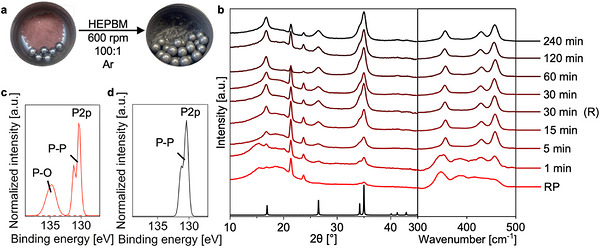
(a) Optimized conditions for the conversion of RP to BP [34], with images of the ball‐milling chamber that show optical transition from red to black. (b) XRD and Raman analyses of samples milled for different durations show the formation of BP. High‐resolution P2p XPS spectra for (c) RP and (d) BP.

In order to achieve a scalable process, a rapid mechanochemical production route that converts red phosphorus (RP) to BP within 30 min (Figure 1a; Table S1) was established. This approach reduces conventional multi‐hour procedures and permits reliable gram‐scale production [34, 35]. The conversion of RP to BP was monitored by Raman, XRD, and UV/Vis spectroscopy. Raman spectra showed the appearance of the characteristic $A_{g}^{1}$ (360 cm^−1^), B_2*g*_ (430 cm^−1^) and $A_{g}^{2}$ (460 cm^−1^) modes after 5 min of milling (Figure 1b). XRD patterns and UV/Vis spectra further confirmed a gradual increase in the BP contribution with no further spectral changes observed after 30 min of milling in reverse mode (30 min R) (Figure 1b; Figure S1). High‐resolution P2p XP spectra of BP, displayed no detectable surface oxidation compared to RP, suggesting that the milling process removes surface oxide species and proceeds under reducing conditions (Figure 1c,d). BP nanosheets (BPNS) were subsequently prepared by liquid‐phase exfoliation. Both exfoliation methods produced BPNS with similar lateral dimensions (Figure S1). To increase the yield for further functionalization, probe sonication was selected, as it produced BPNS with markedly higher yield than bath sonication (Methods section, ESI). XPS confirmed that BPNS produced by both methods were free of surface oxidation and well suited for following covalent functionalization (Figure S1).

### 2.1 Surface Characterization of BPNS Sheets Functionalized via Covalent Triazine Conjugation

**FIGURE 2:**
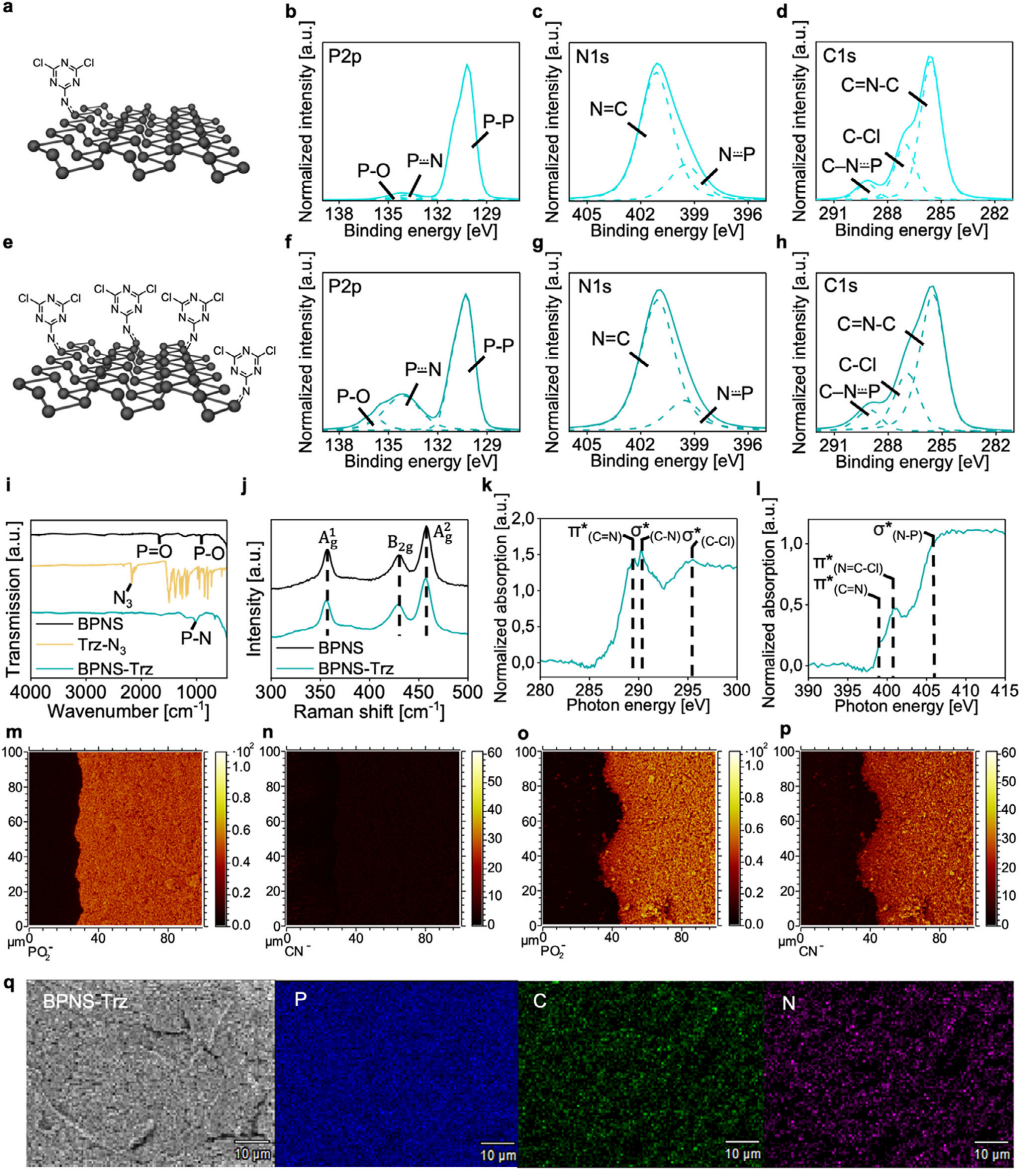
(a) Schematic representation of BPNS‐Trz with lower degree of surface functionalization (without TBAB). High‐resolution (b) P2p, (c) N1s and (d) C1s XP spectra with peak fitting for BPNS‐Trz synthesized without TBAB. (e) Schematic representation of BPNS‐Trz with higher degree of surface functionalization (with TBAB). High‐resolution (f) P2p, (g) N1s and (h) C1s XP spectra with peak fitting for BPNS‐Trz with TBAB. (i) IR comparison of BPNS (black), Trz‐N_3_ (yellow) and BPNS‐Trz (turquoise) with characteristic peaks. (j) Raman comparison of BPNS (black) and BPNS‐Trz (turquoise). NEXAFS spectrum of BPNS‐Trz at the (k) C K‐edge and (l) N K‐edge. ToF‐SIMS images of BPNS fragment distribution of (m) PO_2_^−^ and (n) CN^−^. ToF‐SIMS images of BPNS‐Trz fragment distribution of (o) PO_2_^−^ and (p) CN^−^. (q) SEM‐EDS mapping of the surface of BPNS‐Trz (the scale bar corresponds to 10 µm).

Next, in order to establish a controllable conjugation platform, BPNS were covalently functionalized via a nitrene [2+1] cycloaddition reaction, using a triazine‐azide precursor. Upon activation, the azide generated a reactive nitrene species that covalently attached the 4,6‐dichloro‐1,3,5‐triazine moiety to the BPNS surface (Figure 2a; Table S2). FTIR spectroscopy of 2‐azido‐4,6‐dichloro‐1,3,5‐triazine (Trz‐N_3_) showed the characteristic azide absorption band. Upon reaction with BPNS, this ‐N_3_ band disappeared, and a new signal assigned to P─N bond formation appeared (Figure 2i). Raman spectra of BPNS‐Trz retained three characteristic BP lattice modes with a slight red shift (Figure 2j), which is consistent with covalent functionalization of surface phosphorus atoms and aligns with previous reports on modified BP [36].

Surface sensitive characterization methods collectively confirmed successful covalent conjugation of triazine groups to BPNS and their uniform distribution across the surface. XPS provided direct evidence for covalent conjugation of triazine moieties to BPNS. A N1s photoline in the survey spectrum appeared only after functionalization with triazine (Figure S2). To increase the grafting density, the reaction was also conducted in the presence of the phase‐transfer catalyst tetrabutylammonium bromide (TBAB). Under these conditions, the N1s photoline intensity in the XP survey spectrum increased from ∼3% to ∼7%. The result demonstrated that TBAB significantly enhanced the grafting density (Figure 2a–h; Table S3). Importantly, the C1s and N1s XP spectra of BPNS‐Trz prepared with and without TBAB were nearly identical and confirmed that both routes yield the same triazine modification (Figure 2c,d,g,h). High‐resolution N1s XP spectra exhibited two distinct components assigned to the P─N bond and N─C═N units of the triazine ring. The 1:3 ratio of components corresponded to the chemical structure of the triazine moiety conjugated to BPNS (Figure 2c,g). Consistent with the higher grafting density, the high‐resolution P2p XP spectra showed a significant increase of the P‐N component in the TBAB‐catalyzed sample (Figure 2f). HAXPES, which probes greater sample depths, revealed signals of P1s but not of N1s or C1s. This absence of carbon and nitrogen signals indicated that the triazine groups are only conjugated to the outermost layers of BPNS (Figure S3). NEXAFS at the C and N K‐edges also revealed characteristic absorption features of the dichlorotriazine moieties (Figure 2k,l), that are consistent with previous dichlorotriazine‐functionalized graphene [37]. The N1s σ* (N─P) transition demonstrated that the triazine‐derived nitrogen forms a covalent bond with the BPNS surface (Figure 2l). The morphology and the preservation of the lateral dimensions were confirmed by AFM (Figure S3). ToF‐SIMS detected CN^−^ fragments exclusively for BPNS‐Trz, showing a homogeneous distribution across the surface, which is in good agreement with SEM‐EDS mapping (Figure 2m–q). Further, stability of BP and BPNS‐Trz was systematically compared with XPS under ambient conditions. BP rapidly oxidized within 7 d, while BPNS‐Trz only showed minor oxidation (Figure S4). These results establish a rapid and scalable route of BPNS production process, followed by the covalent triazine functionalization with controllable grafting density. This precise surface chemistry enhances material stability and provides a platform for subsequent post‐modification.

### 2.2 Spectroscopic and Computational Evidence for Monodentate P─N Binding on Functionalized BPNS

**FIGURE 3:**
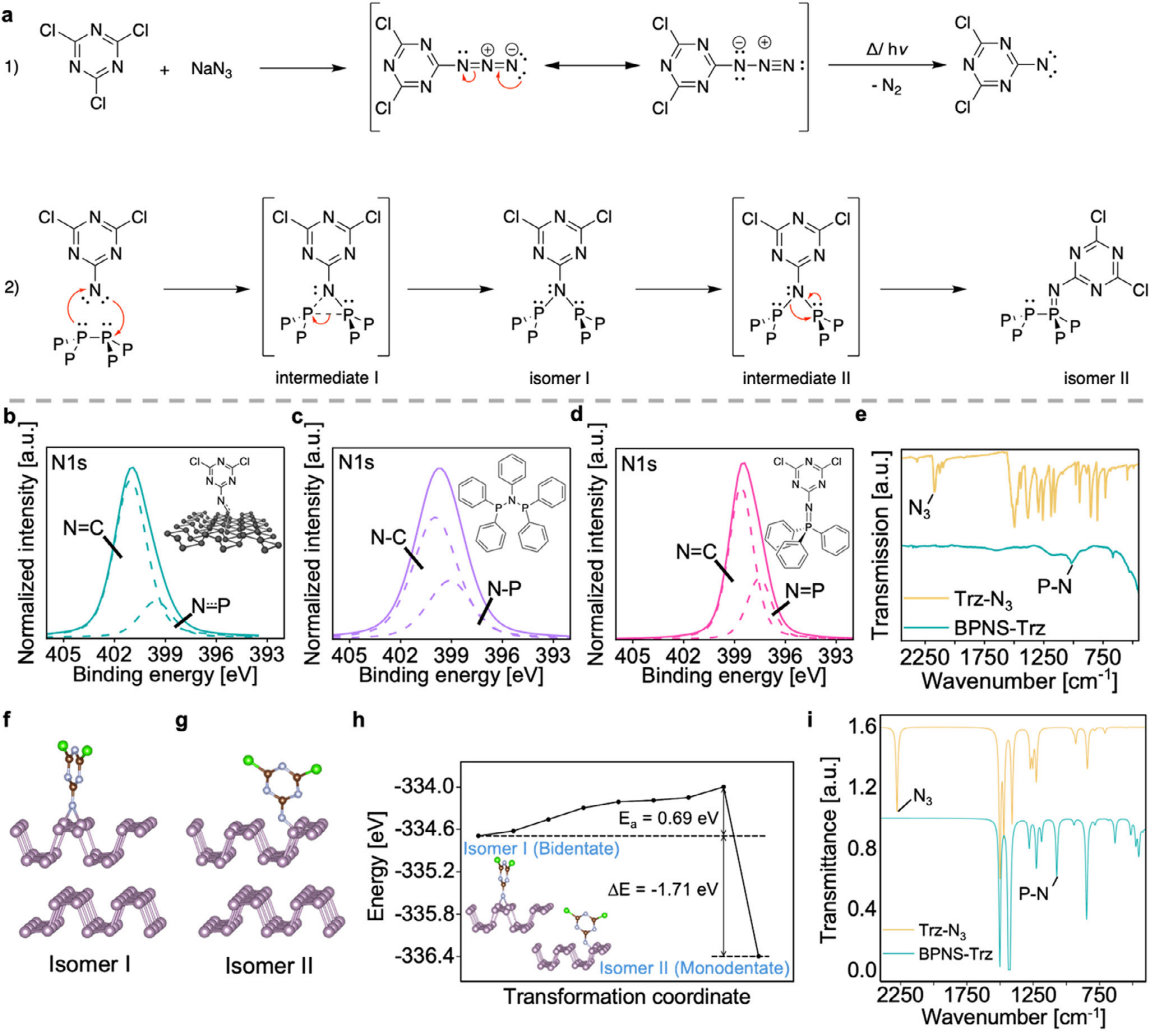
(a) Proposed mechanism for the reaction of BPNS with Trz‐N_3_ [17]. High‐resolution N1s spectra of (b) BPNS‐Trz, (c) high‐resolution N1s of *N*‐(diphenylphosphaneyl)‐*N*‐1,1‐triphenylphosphanamine as a bidentate binding motif, (d) high‐resolution N1s of *N‐*(4,6‐dichloro‐1,3,5‐triazine)triphenylphosphoranylidene as a monodentate binding motif, respectively. (e) IR comparison of Trz‐N_3_ (yellow) and BPNS‐Trz (turquoise) with characteristic peaks. Side view of DFT (PBE+D3) calculations of BPNS‐Trz at low molecular coverage: (f) Isomer I with bidentate P‐N covalent bonds (g) Isomer II with monodentate P‐N covalent bonds. (h) Potential energy landscape of the transformation of isomer I to isomer II. (i) DFPT‐derived IR spectra for Trz‐N_3_ (yellow) and BPNS‐Trz (turquoise).

Two binding motifs have been proposed for nitrene‐derived functionalization of BPNS [17]. First, a bidentate P─N─P diphosphine amine bridge formed through cleavage of a surface P‐P bond (Figure 3a (isomer I)), and second, a monodentate P─N bond that resembles an iminophosphorane‐like environment (Figure 3a (isomer II)). To establish a spectroscopic reference for reliable binding‐mode assignment, reference compounds that represent both configurations were synthesized (N‐(4,6‐dichloro‐1,3,5‐triazine)triphenylphosphoranylidene as a monodentate binding motif; N‐(diphenylphosphaneyl)‐N‐1,1‐triphenylphosphanamine as bidentate binding motif) (Figure 3c,d; Figures S5–S14) [38, 39]. Comparison of the N1s XP spectra revealed that BPNS‐Trz closely matched the monodentate reference compound. At the same time, the bidentate analogue showed pronounced overlapping contributions of N1s components (Figure 3b–d). Due to the presence of only one nitrogen atom in the structure, no stoichiometric N─C/N─P ratio can be defined. In contrast, the monodentate reference (Figure 3d), which contained four nitrogen atoms (as in the conjugated triazine moiety), showed a defined N1s component ratio similar to that observed for BPNS‐Trz. These data indicated that the monodentate configuration is the dominant binding motif on BPNS.

Density functional theory (DFT) calculations were performed to evaluate the relative stability and kinetic accessibility of the two proposed binding motifs on BPNS (Figure 3f–i). At low surface coverage density, both motifs were thermodynamically accessible. However, the monodentate species was thermodynamically strongly favored (ΔE = −2.65 eV vs −0.95 eV for the bidentate isomer I) and formed a short P─N bond (1.62 Å) with partial double‐bond character. To assess the kinetic accessibility of both motifs, potential‐energy calculations revealed a low barrier of 0.69 eV for the transformation of the bidentate isomer into the monodentate isomer. The reverse pathway required 1.71 eV and was therefore inaccessible under the reaction conditions (Figure 3h). Consequently, the monodentate configuration is favored both thermodynamically and kinetically. At higher molecular coverage, the monodentate binding mode remained dominant and showed a slight increase in charge transfer. No pronounced energetic preference for adjacent adsorption was observed and suggested that functionalization is not driven toward clustered binding but occurs in a distributed manner across the surface.

Vibrational analysis further predicted a P‐N stretching mode at 1072 cm^−1^ (Figure 3i; Figure S15), in good agreement with experimental FTIR absorption at 1013 cm^−1^ (Figure 3e). This agreement confirms the formation of covalent P‐N bonds at the BP surface, in parallel with the disappearance of azide vibrational band. Taken together, the comparison with reference materials combined with the vibrational and energetic analyses provided a consistent picture of BPNS‐Trz with a dominant monodentate binding motif.

### 2.3 BP‐Polymer Hybrids as Viral Inhibitors

**FIGURE 4:**
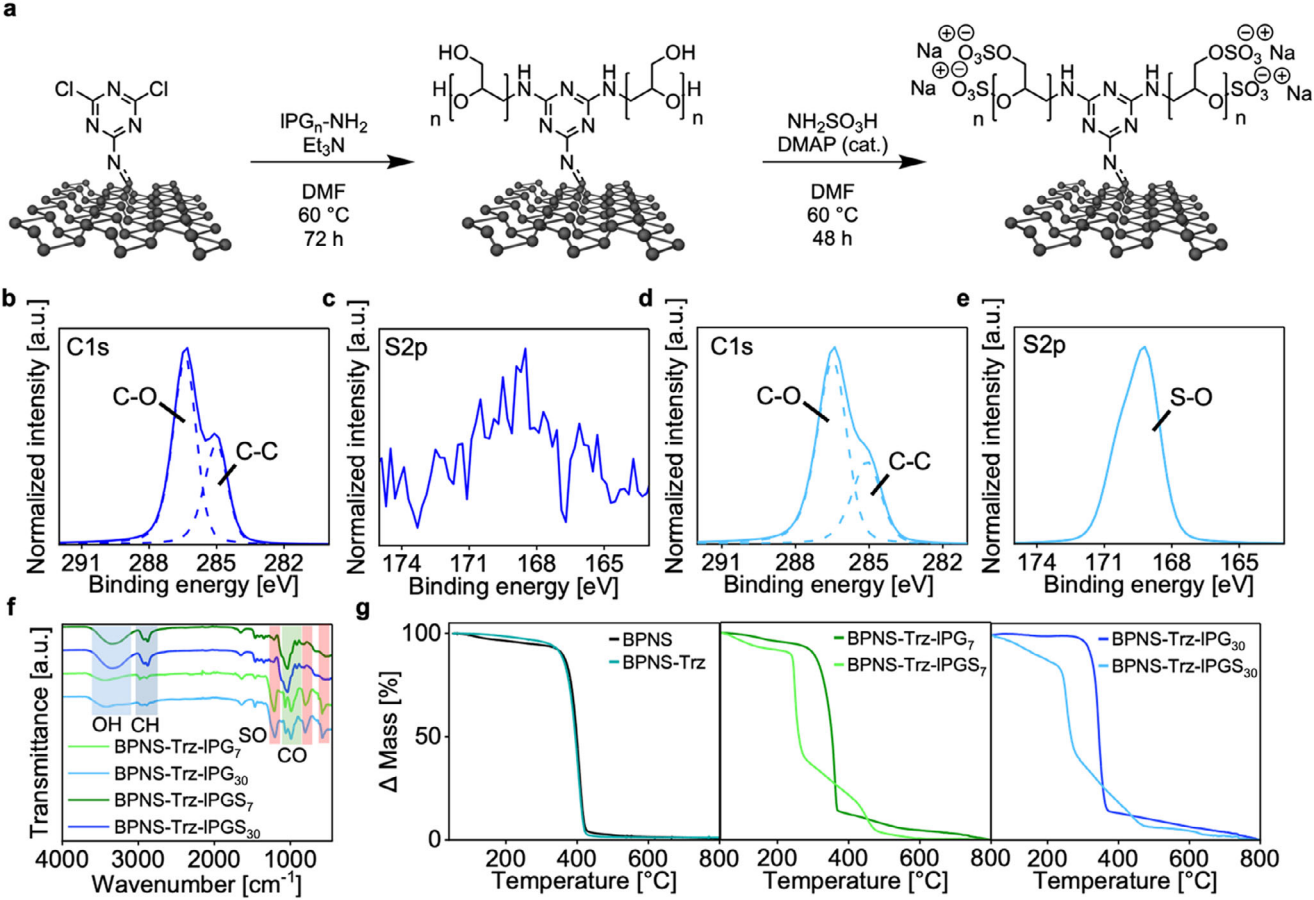
(a) Schematic representation of the synthesis of BPNS‐Trz‐lPGS_n_. High‐resolution (b) C1s with peak fitting and (c) S2p XP spectra for BPNS‐Trz‐lPG_30_, respectively. High‐resolution (d) C1s and (e) S2p XP spectra with peak fitting for BPNS‐Trz‐lPGS_30_, respectively. (f) Comparison of FTIR spectra of BPNS‐Trz‐lPG_7_ (dark green), BPNS‐Trz‐lPG_30_ (dark blue), BPNS‐Trz‐lPGS_7_ (light green) and BPNS‐Trz‐lPG_30_ (light blue) including characteristic peak assignments. (g) TGA curve comparison of BPNS (black), BPNS‐Trz (turquoise), BPNS‐Trz‐lPG_7_ (dark green), BPNS‐Trz‐lPGS_7_ (light green), BPNS‐Trz‐lPG_30_ (dark blue) and BPNS‐Trz‐lPGS_30_ (light blue).

Aminated lPG precursors of two molecular weights (7 and 30 kDa) were conjugated to BPNS‐Trz via nucleophilic aromatic substitution of chlorines on the dichlorotriazine moiety (Figure 4a). The reaction yielded water‐dispersible BPNS‐Trz‐lPG_7/30_ conjugates. FTIR spectroscopy and zeta potential measurements indicated polymer attachment, and TGA revealed a significant change in thermal behavior (Figure 4f,g). Together, these results confirm successful functionalization. Following subsequent sulfation reaction, BPNS‐Trz‐lPGS_7_ and BPNS‐Trz‐lPGS_30_ were obtained, which displayed high sulfur content and strongly negative zeta potentials (Table S2). Both findings indicate successful introduction of sulfate groups. Successful polymer conjugation and sulfation were further confirmed by surface‐sensitive analyses. XPS revealed characteristic C1s peak components of the polyglycerol backbone, while S2p signals verified successful sulfation of the polymer (Figure 4b–e). In agreement with dense polymer surface coverage and limited probing depth of XPS, phosphorus was not detectable (Figure S16). FTIR spectra showed expected C─O stretching modes of lPG, along with S─O and C─O─S bands associated with sulfation (Figure 4f). Thermogravimetric analysis (TGA) further supported successful sulfation through a pronounced decrease in thermal stability (Figure 4g), consistent with reported behavior of sulfated materials [25, 40].

**FIGURE 5:**
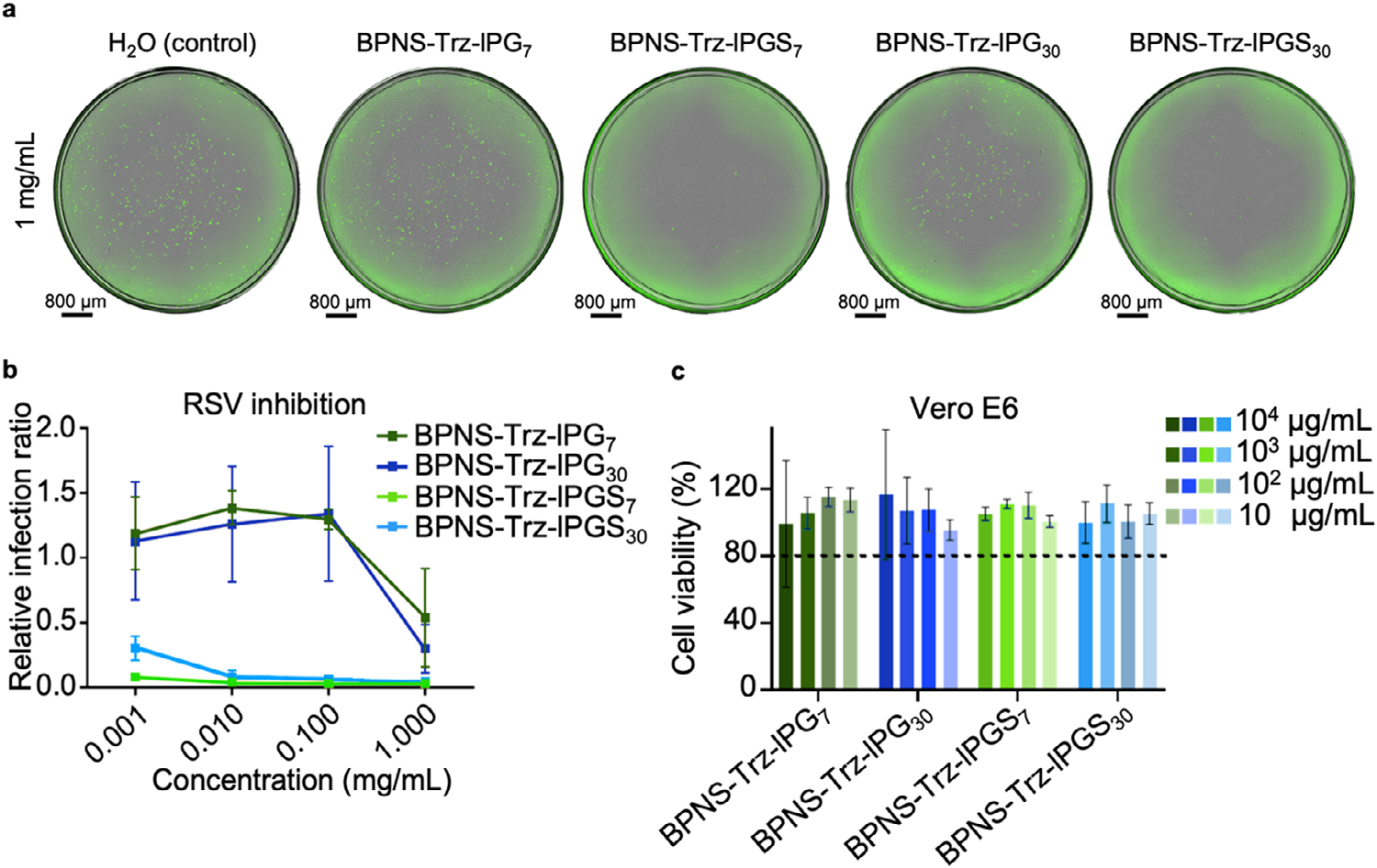
(a) Comparison of inhibitors at 1 mg/mL concentration against RSV‐GFP infection on A549 cells. Scale bar: 800 µm. (b) Quantification of the infection reduction of non‐sulfated and sulfated compounds against RSV‐GFP. (c) Cell viability of produced compounds at various concentrations on Vero E6 cells.

The antiviral activity of sulfated BP‐polymer hybrids was subsequently evaluated in cell‐based infection assays. BPNS‐Trz‐lPGS_7/30_ conjugates showed strong antiviral activity against RSV. In infection reduction assays using a RSV reporter virus with green fluorescent protein (RSV‐GFP) [41], in A549 cells, BPNS‐Trz‐lPGS_7_ and BPNS‐Trz‐lPGS_30_ showed strong inhibition at a concentration of 1 mg/mL. Pronounced antiviral activity was maintained even at much lower concentration of 0.01 mg/mL. Dose‐response analysis yielded a half‐maximal inhibitory concentration (IC_50_) of ∼0.02 µg/mL for BPNS‐Trz‐lPGS_7_ and ∼0.37 µg/mL for BPNS‐Trz‐lPGS_30_ on RSV‐GFP. In contrast, non‐sulfated BP‐polymer precursors as well as the sulfated polymers alone showed little to no inhibition across all tested concentrations (Figure 5a,b; Figures S17 and S18). Similar trends were observed against HSV‐1, where the sulfated materials reduced viral infection in plaque reduction assays (Figure S16). However, in contrast to RSV, strong inhibition was observed only for BPNS‐Trz‐lPGS_7_, whereas BPNS‐Trz‐lPGS_30_ showed no detectable antiviral activity. Together, these results indicate that shorter polymer chains consistently outperform longer ones across both viral systems. This behavior suggests that antiviral efficacy correlates with the surface charge density, as reflected by the more negative zeta potential of BPNS‐Trz‐lPGS_7_. Shorter polymer chains may enable a higher density of accessible sulfate groups at the BP surface and promote stronger electrostatic interactions with viral surface proteins, rather than charge transfer processes, similar to the previously reported systems [42]. Cytotoxicity experiments with Vero E6 cells showed >80% viability across all tested concentrations (10–10^4^ µg/mL) (Figure 5c). In summary, these results demonstrated that BP‐lPGS hybrids act as multivalent anionic inhibitors that can interfere with viral surface proteins through electrostatic interactions and lead to strong inhibition of both HSV‐1 and RSV.

## 3 Conclusion

In conclusion, we introduce a scalable covalent functionalization strategy for BP with well‐defined triazine surface chemistry. The triazine‐based functionalization of BP enables controlled attachment of nucleophiles through substitution reactions. Post‐modification of functionalized BP with linear polyglycerol derivatives provides a clear example of this tunable approach to design BP‐based hybrid materials. Antiviral studies provide proof of concept that sulfated BP‐polymer hybrids interfere with viral entry pathways in vitro. Together with the rapid and scalable mechanochemical production of BP, these findings underscore the broader utility of this covalent functionalization strategy toward chemically controlled and biodegradable 2D nanomaterials for biomedical applications.

## Author Contributions

J.E.: investigation, methodology, validation, formal analysis, writing – original draft. S.R.: investigation, formal analysis. E.X.: investigation, formal analysis. N.X.: investigation, methodology, formal analysis. R.S.: investigation, formal analysis. A.W.: investigation. M.R.: investigation. T.P.: investigation. R.E.: investigation. P.N.: investigation, formal analysis. R.A.: investigation, formal analysis. A.H.: investigation, methodology. O.B.: methodology. A.H.: supervision. J.R. and V.‐D.H.: investigation. C.S.: supervision, methodology, and funding acquisition. B.P.: supervision, methodology, funding acquisition. I.S.D.: conceptualization, supervision, methodology, project administration, funding acquisition, writing – review, and editing.

## Funding

This work is supported by the NanoMatFutur (PathoBlock, project number 13XP5191), China Scholarship Council (Grant No. 202106990020), and Helmholtz Association (VH‐NG‐1526).

## Conflicts of Interest

The authors declare no conflicts of interest.

## Supporting information




**Supporting File**: smll73572‐sup‐0001‐SuppMat.docx.

## Data Availability

The data that support the findings of this study are available from the corresponding author upon reasonable request.
