# Supplementary material for: Controlled Triazine‐Based Covalent Functionalization of Black Phosphorus for Degradable Hybrid Materials
Source: Small. 2026 Apr 27;22(34):e73572. doi: 10.1002/smll.73572 (PMC13275033; doi:10.1002/smll.73572)
Supplement: Supplementary file 1 — Supporting File: smll73572‐sup‐0001‐SuppMat.docx. [file SMLL-22-e73572-s001.docx]

Supplementary Information

**Controlled Triazine-Based Covalent Functionalization of Black Phosphorus for Degradable Hybrid Materials**

*Jasmin Er^ab^, Sreejita Ray^c^, Enyu Xie^d^, Robert Schusterbauer^ab^, Maik Rosentreter^a^, Ranen Etouki^a^, Na Xing^a^, Anja Wiesner^a^, Philip Nickl^ab^, Rameez Ahmed^a^, Taylor Page^a^, Ana Hočevar^a^, Obida Bawadkji^a^, Andreas Herrmann^a^, Jörg Radnik^b^, Vasile-Dan Hodoroaba^b^, Christian Sieben^d^, Beate Paulus^c^, Ievgen S. Donskyi^ab*^*

^a^Institut für Chemie und Biochemie, Freie Universität Berlin, Takustraße 3, 14195 Berlin, Germany

^b^Federal Institute for Material Research and Testing (BAM), Division 6.1, Unter den Eichen 44-46, 12203 Berlin, Germany ^c^Institut für Chemie und Biochemie, Physikalische und Theoretische Chemie, Freie Universität Berlin, Arnimallee 22, 14195 Berlin, Germany

^d^Nanoscale Infection Biology Group, Helmholtz Centre for Infection Research, Inhoffenstr. 7 38124 Braunschweig, Germany

*Corresponding author. E-mail: ievgen.donskyi@fu-berlin.de

Table of contents

[1. Experimental Section 3](#_Toc227751856)

[1.1. Materials and methods 3](#_Toc227751857)

[1.1.1. Reaction conditions and chemicals 3](#_Toc227751858)

[1.1.2. High Energy Planetary Ball-Mill (HEPBM) 4](#_Toc227751859)

[1.1.3. Nuclear magnetic resonance (NMR) spectroscopy 4](#_Toc227751861)

[1.1.4. Raman spectroscopy 4](#_Toc227751863)

[1.1.5. Infrared (IR) spectroscopy 5](#_Toc227751864)

[1.1.6. X-ray photoelectron spectroscopy (XPS) 5](#_Toc227751865)

[1.1.7. Hard X-ray photoelectron spectroscopy (HAXPES) 6](#_Toc227751866)

[1.1.8. Near edge X-ray adsorption fine structure (NEXAFS) 6](#_Toc227751867)

[1.1.9. Time-of-flight secondary mass spectrometry (ToF-SIMS) 6](#_Toc227751868)

[1.1.10. Zeta-potential 7](#_Toc227751869)

[1.1.11. Thermogravimetric analysis (TGA) 7](#_Toc227751870)

[1.1.12. Atomic force microscopy (AFM) 7](#_Toc227751871)

[1.1.13. Cell culture and virus strains 8](#_Toc227751872)

[1.1.14. Cell viability assays 8](#_Toc227751873)

[1.1.15. Plaque reduction assay HSV-1 8](#_Toc227751874)

[1.1.16. Infection assay with RSV-GFP 9](#_Toc227751875)

[1.1.17. Pre-infection inhibition assay 9](#_Toc227751876)

[1.2. Synthesis 10](#_Toc227751877)

[1.2.1. Screening experiment to produce black phosphorus (BP) 10](#_Toc227751878)

[1.2.2. Liquid phase exfoliation (LPE) of BP with an ultrasonic bath 11](#_Toc227751881)

[1.2.3. LPE of BP with probe sonication 11](#_Toc227751882)

[1.2.4. Synthesis of BPNS-Trz 11](#_Toc227751883)

[1.2.5. Synthesis of 2-azido-4,6-dichloro-1,3,5-triazine 12](#_Toc227751885)

[1.2.6. Synthesis of 3,5-dichloro-*N-*(triphenylphosphoranylidene)aniline 12](#_Toc227751887)

[1.2.7. Synthesis of *N-*(4,6-dichloro-1,3,5-triazine)triphenylphosphoran-ylidene 13](#_Toc227751888)

[1.2.8. Synthesis of N-(diphenylphosphaneyl)-N-1,1-triphenylphosphanamine 14](#_Toc227751890)

[1.2.9. Synthesis of BPNS-triazine linear-polyglycerol (BPNS-Trz-lPG_n_) 15](#_Toc227751892)

[1.2.10. Synthesis of BPNS-triazine linear-polyglycerol-sulfate (BPNS-Trz-lPGS_n_) 15](#_Toc227751902)

[2. Supplementary figures and tables 16](#_Toc227751910)

[**Table S1**. Ball-milling parameters for the synthesis of BP 10](#_Toc227751911)

[**Table S2**. Characterization of BP derivatives starting from pristine BP 16](#_Toc227751911)

[**Figure S1.** Characterization of BP and BPNS 17](#_Toc227751912)

[**Figure S2.** XP survey spectra for BPNS and BPNS-Trz 18](#_Toc227751913)

[**Table S3.** Relative elemental fractions from the quantification of the XPS survey 18](#_Toc227751914)

[**Figure S3.** Characterization of BPNS-Trz 19](#_Toc227751915)

[**Figure S4.** Stability experiments for the degradation of BP and BPNS-Trz 21](#_Toc227751916)

[**Figure S5.** IR comparison of synthesized P=N/P-N control materials. 22](#_Toc227751917)

[**Figure S6.** ^1^H-NMR of 3,5-dichloro-*N-*(triphenylphosphoranylidene)aniline. 22](#_Toc227751918)

[**Figure S7.** ^31^P-NMR of 3,5-dichloro-*N-*(triphenylphosphoranylidene)aniline. 23](#_Toc227751919)

[**Figure S8.** ESI of 3,5-dichloro-*N-*(triphenylphosphoranylidene)aniline. 23](#_Toc227751920)

[**Figure S9.** ^1^H-NMR of *N-*(4,6-dichloro-1,3,5-triazine)triphenylphosphoranylidene. 24](#_Toc227751921)

[**Figure S10.** ^31^P-NMR of *N-*(4,6-dichloro-1,3,5-triazine)triphenylphosphoranylidene. 24](#_Toc227751922)

[**Figure S11.** ESI of *N-*(4,6-dichloro-1,3,5-triazine)triphenylphosphoranylidene. 25](#_Toc227751923)

[**Figure S12.** ^1^H-NMR of *N*-(diphenylphosphaneyl)-*N*-1,1-triphenylphosphanamine. 25](#_Toc227751924)

[**Figure S13.** ^31^P-NMR of *N*-(diphenylphosphaneyl)-*N*-1,1-triphenylphosphanamine. 26](#_Toc227751925)

[**Figure S14.** ESI of *N*-(diphenylphosphaneyl)-*N*-1,1-triphenylphosphanamine. 26](#_Toc227751926)

[**Figure S15.** Γ-point phonon frequencies 27](#_Toc227751927)

[**Figure S16.** Characterization of BPNS-Trz-lPG/Sn 29](#_Toc227751928)

[**Figure S17.** RSV-GFP inhibition of BP-hybrids 30](#_Toc227751929)

[**Figure S18.** RSV-GFP inhibition of sulfated polymers 31](#_Toc227751930)

[**Figure S19.** ^1^H-NMR (500 MHz) spectrum of BP-Trz-lPG_7_ in D_2_O. 32](#_Toc227751931)

[**Figure S20.** ^1^H-NMR (500 MHz) spectrum of BP-Trz-lPG_30_ in D_2_O. 32](#_Toc227751932)

[**Figure S21**. ^1^H-NMR (500 MHz) spectrum of BP-Trz-lPGS_7_ in D_2_O. 33](#_Toc227751934)

[**Figure S22.** ^1^H-NMR (500 MHz) spectrum of BP-Trz-lPGS_30_ in D_2_O. 33](#_Toc227751935)

[**Figure S23.** ^13^C-NMR (125 MHz) spectrum of BP-Trz-lPG_7_ in D_2_O. 34](#_Toc227751937)

[**Figure S24.** ^13^C-NMR (125 MHz) spectrum of BP-Trz-lPG_30_ in D_2_O. 34](#_Toc227751939)

[3. References 35](#_Toc227751940)

# Experimental Section

## Materials and methods

### Reaction conditions and chemicals

Unless otherwise stated all reactions were conducted under an inert argon atmosphere using schlenk techniques or in an inert glovebox. Red phosphorus (≥97.0%, SigmaAldrich), sodium azide (≥99.0%, NaN_3_, Alfa Aesar), cyanuric chloride (≥99.0%, SigmaAldrich), tetrabutylammonium bromide (≥99.0%, TBAB, SigmaAldrich), triethylamine (≥99.5%, Et_3_N, SigmaAldrich), sulfamic acid (SigmaAldrich), 4‑(dimethylamino)pyridine (DMAP, SigmaAldrich), sodium hydroxide (≥98.0%, Fisher Scientific), chlorodiphenylphosphine (98.0%, Fisher Scientific), triphenylphosphine (≥99.0%, SigmaAldrich), 3,5-dichloroaniline (≥98.0%, Fisher Scientific), aniline (≥99.9%, Acros organics) were purchased from commercial suppliers and used without further purification. lPGs with an amine end group (7 kDa and 30 kDa) were synthesized and provided by Daniel Kutifa (Freie Universität Berlin). Dry NMP, IPA and DMF were purchased from Acros organics and used as received unless otherwise stated. For all reactions that involved air- or water-sensitive compounds the solid reagents were dried on the schlenk line (1·10^-3^ mbar) at least one day prior use. Water was used from Mili-Q^®^ Advantage A10 Water Purification System. Purification was performed in Spectra/Por^®^ 6 cellulose dialysis tubes (100 kDa, 50 kDa and 2 kDa MWCO, Repligen).

### High Energy Planetary Ball-Mill (HEPBM)

The Planetary Mono Mill Pulverisette 6 *classic line* from Fritsch GmbH (Germany) was used for the conversion process. The 80 mL agate grinding bowl (Fritsch GmbH, Germany) made of hardened stainless steel was only opened inside the glovebox and cleaned after every use using dry IPA. Stainless steel balls with a diameter of 10 mm were used (Fritsch GmbH, Germany). Milling time, pause time and number of repetitions was varied. All experiments were performed at an rpm of 600.

### Nuclear magnetic resonance (NMR) spectroscopy

NMR measurements were performed on a Joel ECZ600 (Japan) and a Bruker AVANCE III 500 (USA). Processing of the data was done using the software MestReNova (version 14.3.0). Chemical shifts δ were given in ppm in relativity to an internal standard, usually the deuterated solvent D_2_O (δ (1H) = 4.79 ppm) or CDCl_3_ (δ (1H) = 7.26 ppm).

### Raman spectroscopy

Raman spectroscopy was conducted using a Horiba XploRA Plus**™** spectrometer (Japan), equipped with a 532 nm Nd:YAG laser and a motorized x/y piezo stage. The laser beam was focused through a 100x Nikon® objective lens, with prepared samples being irradiated at 1 mW (1% of 100 mW, with the actual energy reduced by a filter). The spectrometer, which was autocalibrated with a silicon wafer prior to measurements, was set to a 2400 cm⁻¹ grating centered at 600 cm⁻¹. The samples were deposited on 1 cm x 1 cm silicon wafers. For statistical raman spectroscopy, raman maps were generated from selected sample regions using the point by point method, with substrate movement during mapping controlled by the motorized x/y piezo stage. Average spectra were created from these maps. To analyze the data, a polynomial baseline fit was applied in LabSpec 6 (HORIBA).

### Infrared (IR) spectroscopy

IR spectra were recorded using a Spectrum Two FT-IR spectrometer by PerkinElmer (USA). The measuring range was set between 4000 cm^-1^ to 450 cm^-1^. Samples were measured at room temperature by adding small amounts of sample to the crystal.

### X-ray photoelectron spectroscopy (XPS)

XPS measurements were performed using an EnviroESCA spectrometer (SPECS Surface Nano Analysis GmbH, Berlin, Germany), equipped with a monochromatic Al Kα X-ray source (Excitation Energy = 1486.71 eV) and a PHOIBOS 150 electron energy analyzer operating in fixed analyzer transmission (FAT) mode. All spectra were acquired under ultra-high vacuum. Samples for XPS analysis were prepared on silicon wafers or indium foil. The spectra were measured in normal emission, and a source-to-sample angle of 55° was used. Instrument calibration followed the technical procedure provided by SPECS (calibration was performed according to ISO 15472). Survey spectra were acquired with a pass energy of 80 electron volt (eV), and the high-resolution XP spectra were acquired with a pass energy of 50 eV. Raw data fitting was performed using the UNIFIT 2022 software. For fitting, a Shirley background and a Lorentzian-Gaussian (L-G) sum function were used. Unless stated otherwise, a L-G mixing ratio of 0.3 was used for carbon peaks and 0.40 for heteroatom peaks. If not indicated otherwise, all binding energies were referenced to the binding energy of sp^3^-hybridized C–C bond component at 285 eV or the energy of C-N=C at 285.5 eV.

### Hard X-ray photoelectron spectroscopy (HAXPES)

HAXPES experiments were carried out using a monochromatic Cr Kα source (5.4 keV, Ulvac-PHI, Chanhassan, USA). For charge neutralization, low-energy electrons and Ar^+^-ions were employed. The utilized spot size was 100 µm and photoelectrons were detected at an emission angle of 45° relative to the surface. Measurements were performed at pressures between 10^-8^ and 10^-10^ mbar, with samples mounted to the sample holder using adhesive tape. High-resolution HAXPES spectra were measured using a pass energy of 55 eV, a step size of 0.1 eV and 10-20 sweeps, depending on the signal-to-noise ratio of each element.

### Near edge X-ray adsorption fine structure (NEXAFS)

NEXAFS experiments were carried out at the synchrotron radiation source BESSY II (Berlin, Germany) at the HE-SGM monochromator dipole magnet CRG beamline. NEXAFS spectra were acquired in total energy electron yield (TEY) mode using a channel plate detector. The resolution E/ΔE of the monochromator at the carbonyl π* resonance (hν = 287.4 eV) was in the order of 2500. Raw 3 spectra were divided by ring current and monochromator transmission, the latter obtained with a freshly sputtered Au sample. Alignment of the energy scale was achieved by using an I_0_ feature referenced to a C1s → π* resonance at 285.4 eV measured with a fresh surface of HOPG (highly ordered pyrolytic graphite, Advanced Ceramic Corp., Cleveland, USA). If not otherwise denoted, all NEXAFS spectra are shown after subtraction of the pre-edges followed by normalization of the post-edge count rates to one. All C K-edges were measured at 55° incident angle of the linearly polarized synchrotron light.

### Time-of-flight secondary mass spectrometry (ToF-SIMS)

ToF-SIMS analysis was performed using a TOF-SIMS M6 instrument (IONTOF GmbH, Münster, Germany). The samples were analyzed at room temperature. Measurements were conducted in fast imaging mode with a 30 keV Bi₃⁺ primary ion beam in negative polarity. A field of view (FoV) measuring 100 × 100 μm was rastered in random mode with 2048 × 2048 pixels, acquiring one shot per pixel. All images were binned by a factor of 16. Spectra were calibrated using typical organic fragments (C^-^ at 12.00, C₂^-^ at 24.00, C_3_^-^ at 36.00, C_4_^-^ at 48.00 m/z).

### Zeta-potential

Zeta-potential measurements were carried out in Milli-Q water at ambient conditions using a Zetasizer Ultra (Malvern Panalytical, UK). Samples were analyzed in a folded capillary zeta cell (Malvern Panalytical, UK) operated in automatic mode. Each measurement was repeated three times and the average zeta-potential value was reported.

### Thermogravimetric analysis (TGA)

TGA experiments were performed on a TGA 8000 (PerkinElmer, USA) under a nitrogen atmosphere with a nitrogen flow of 20 ml min^-1^. Samples containing black phosphorus were purged with nitrogen prior measurement for 1 h. The heating rate was set to 10 °C min^-1^ and the instrument was calibrated using calcium oxalate. Al_2_O_3_ crucibles were used and the sample mass varied from 1 to 3 mg.

### Atomic force microscopy (AFM)

All samples were imaged with a JPK nanowizard AFM (Bruker) with in AC mode (also known as tapping mode). All experiments were performed in air and samples were prepared as follows. Rectangular pieces of muscovite mica or silicon wafers of about 1 cm^2^ were used as a substrate. Double sided tape was used to glue the mica to circular metal pucks. It was then cleaved with regular adhesive tape to obtain a clean flat surface. 10 µl of the samples dispersed in water or MeOH (Concentration: 0.1 mgml^-1^) was placed in the middle and allowed to spread and eventually dry on the surface. The samples were then mounted on the XY stage. TAP300Al-G silicon AFM probes from Budget sensors with a nominal spring constant of 40 N m^-1^ and tip radius of less than 10 nm were used. Scan rates were usually 1 Hz with 256 points per line. The JPKSPM data processing software Version 6.1.74 (trademark JPK instruments) was used for Image analysis. In images obtained with sheets, a XY plane fit order 1 was performed, followed by a flatten order 1 as well. Determination of sheet thickness was done using the cross-section analysis function.

### Cell culture and virus strains

A549 human lung carcinoma cells (DSMZ, ACC 107), Hep-2 cells (ATCC, CCL-23) and Vero E6 cells (ATCC CCL-81) were cultured in Dulbecco’s modified Eagle’s medium (DMEM) supplemented with 10% fetal bovine serum (FBS), 100 U mL^-1^ penicillin, and 100 μg mL^-1^ streptomycin. All cells were cultured at 37°C and 5% CO_2_.

The HSV-1 strain F carrying GFP (kindly provided by Y. Kawaguchi, University of Tokyo, Japan) was propagated on Vero E6 cells. RSV-GFP^[1]^ (human respiratory syncytial virus subtype A Long strain carrying a GFP reporter gene) was propagated in Hep-2 cells.

### Cell viability assays

To analyze the cytotoxicity and determine the half-maximal cytotoxic concentration (CC50) of compounds, the CellTiter-Glo® Luminescent Cell Viability Assay Kit (Progema Corporation) was used according to the manufacturer’s instructions. Briefly, cells were seeded in an opaque-walled 96-well plate and cultured in a CO_2_ incubator at 37°C for 24 h. After overnight incubation, various concentrations of compounds ranging from 0.1-1000 μg mL^-1^ were added to the cells and incubated for another day Then, equal amount of CellTiter-Glo® Reagent was added to the cell culture medium and mixed for 2 minutes on an orbital shaker to induce cell lysis. At room temperature the plate was incubated for 10 minutes to stabilize the luminescent signal. Subsequently, the luminescence was determined by a microplate reader with the integration time of 0.25-1 second/well.

### Plaque reduction assay HSV-1

10-fold serially diluted samples were incubated with 100 plaque forming unit (PFU) of viruses for 45 min at 37 **°**C. Then, the viruses and compounds mixture were added on a confluent monolayer of cells. Virus adsorption was allowed for 1 h before the supernatant was aspirated and replaced by a semi-solid overlay. After 48-72h, the overlay was removed, and cells were fixed with 4% formaldehyde solution and stained with 0.1% crystal violet. The percentage of plaque reduction was calculated by the plaque numbers of virus control and samples.

### Infection assay with RSV-GFP

A549 cells were seeded in 96-well plates at a density of 2 × 10^4 cells per well and incubated overnight at 37 °C in 5% CO_2_. 3,600 PFU of RSV-GFP^[1]^ was pre-incubated with the indicated concentrations of compounds for 45 min at room temperature and then added to the cells. After 2 h, the inoculum was removed and replaced with infection medium (DMEM supplemented with 2% FBS), and the cells were further cultured at 37 °C in 5% CO_2_. At 24 h post-infection, cells were imaged, and the number of infected cells was quantified using an Incucyte live-cell imaging system (IncuCyte S3, Sartorius, Germany). RSV-GFP incubated with double-distilled water (ddH_2_O; solvent of the compounds) served as the control group. Antiviral activity was expressed as the relative inhibition ratio, calculated by comparing the number of infected cells in compound-treated wells with that in control wells.

### Pre-infection inhibition assay

Cells grown in a 96-well plate at 100% confluence were treated with various concentrations of antiviral compounds (ranging from 0.1 μg mL^-1^ to 1 mg mL^-1^) for 45 min at 37 °C with continuously shaking and then infected by viruses at a MOI of 0.01 for 48 h. Afterwards, HSV-1 infected cells were stained with Hoechst 33342 and fixed with 4% formaldehyde in phosphate-buffered saline (PBS) for 30 min. For 229E or FCoV pre-infection assay, infected cells were fixed and permeabilized with 0.1% Triton X-100 in PBS for 10 min, and blocked with 3% bovine serum albumin (BSA) in PBS for 30 min. Cells were then incubated with primary antibodies (mouse anti-IBV monoclonal antibody provided by Gary R. Whittaker or Rabbit anti-229E polyclonal antibody from Bio-Rad) for 1 h, washed three times with PBS, and stained with secondary antibodies anti-mouse Alexa Fluor 488 fluorophores or anti-rabbit Alexa Fluor 594 fluorophores (Thermo Fisher Scientific) for 1 h with gentle shaking. The cell nucleus was stained with Hoechst 33342. Images were acquired using Zeiss fluorescent microscope and the percentage of infection inhibition were analyzed.

## Synthesis

### Screening experiment to produce black phosphorus (BP)

**
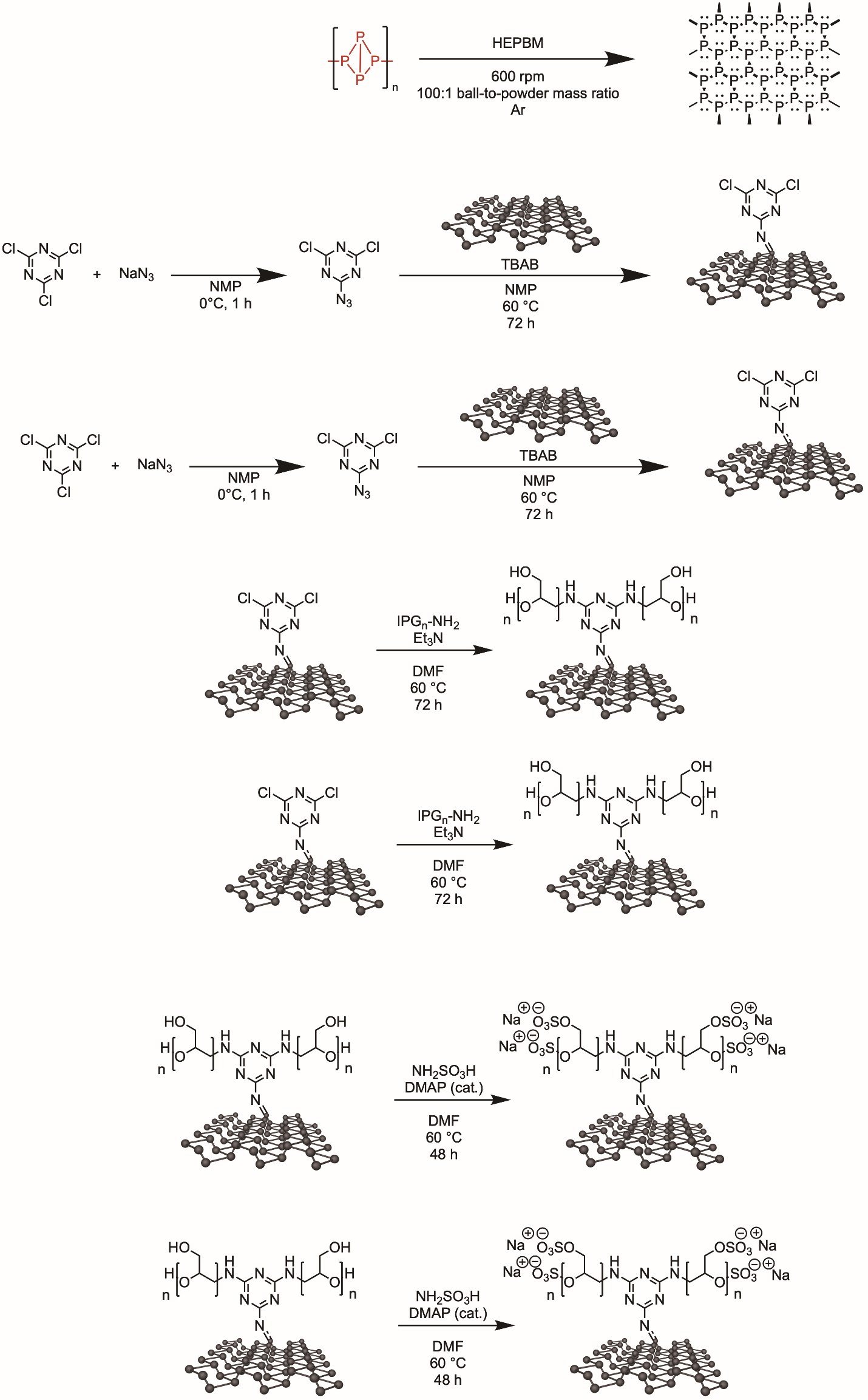
**

**Scheme S1.** Scheme for the production of BP.

Inside a glovebox, dried RP (1.00 g) was transferred into an 80 mL agate milling chamber containing 25 steel balls (~100 g). The chamber was sealed under an argon atmosphere using a screw clamp system to maintain the inert environment and subsequently placed on the Pulverisette 6. Milling was conducted at a rotational speed of 600 rpm under various milling duration, pause intervals and cycle repetitions (table S1). In runs, were pause times where applied, the rotation direction was reversed after each pause to promote more uniform conversion. Upon completion, the resulting BP was collected inside the glovebox.

**Table S1.** Ball milling parameters for the synthesis of BP, including milling time, pause times, number of cylcles, reverse mode and total milling time.

| Milling time per Cycle  [min] | Pause time  [min] | Cycles | Reverse mode after pause | Total milling time  [min] |
| --- | --- | --- | --- | --- |
| 1 | - | 1 | No | 1 |
| 5 | - | 1 | No | 5 |
| 15 | - | 1 | No | 15 |
| 15 | 15 | 2 | Yes | 30 |
| 30 | - | 1 | No | 30 |
| 30 | 15 | 1 | Yes | 60 |
| 30 | 30 | 4 | Yes | 120 |
| 30 | 30 | 8 | Yes | 240 |

### Liquid phase exfoliation (LPE) of BP with an ultrasonic bath

Inside a glovebox, bulk BP (1.00 g) was placed inside a 250 mL round bottom flask and sealed before removal from the argon atmosphere. NMP (100 mL) was added to reach a concentration of 10 mg mL^-1^. The suspension was sonicated in a bath sonicator operating at full power for 8 h at room temperature. After, the dispersion was divided between two centrifuge tubes and subjected to centrifugation (45 min, 1500 rpm, 4 °C) to remove bulky material. The upper 2/3 were collected. To purify the BPNS, the supernatant was further centrifuged (6 x 15 min, 9500 rpm, 4 °C) and redispersed in acetone between each cycle. The final product was obtained as a black powder after lyophilization (~10 mg) and stored under argon atmosphere.

### LPE of BP with probe sonication

Inside a glovebox, bulk BP (1.00 g) was placed inside a 100 mL round bottom flask and sealed before removal from the argon atmosphere. NMP (100 mL) was added to achieve a concentration of 10 mg mL^-1^. The suspension was sonicated using a tip sonicator submerged directly into the dispersion operated at full power for 1 h under ice cooling. The resulting dispersion was transferred into two centrifuge tubes and subjected to centrifugation (45 min, 1500 rpm, 4 °C) to remove bulky material. The upper 2/3 were collected. To purify the BPNS, the suspension was further centrifuged (6 x 15 min, 9500 rpm, 4 °C) and redispersed in acetone between each cycle. The final product was obtained as a black powder after lyophilization (~100 mg) and stored under argon atmosphere.

### Synthesis of BPNS-Trz

**
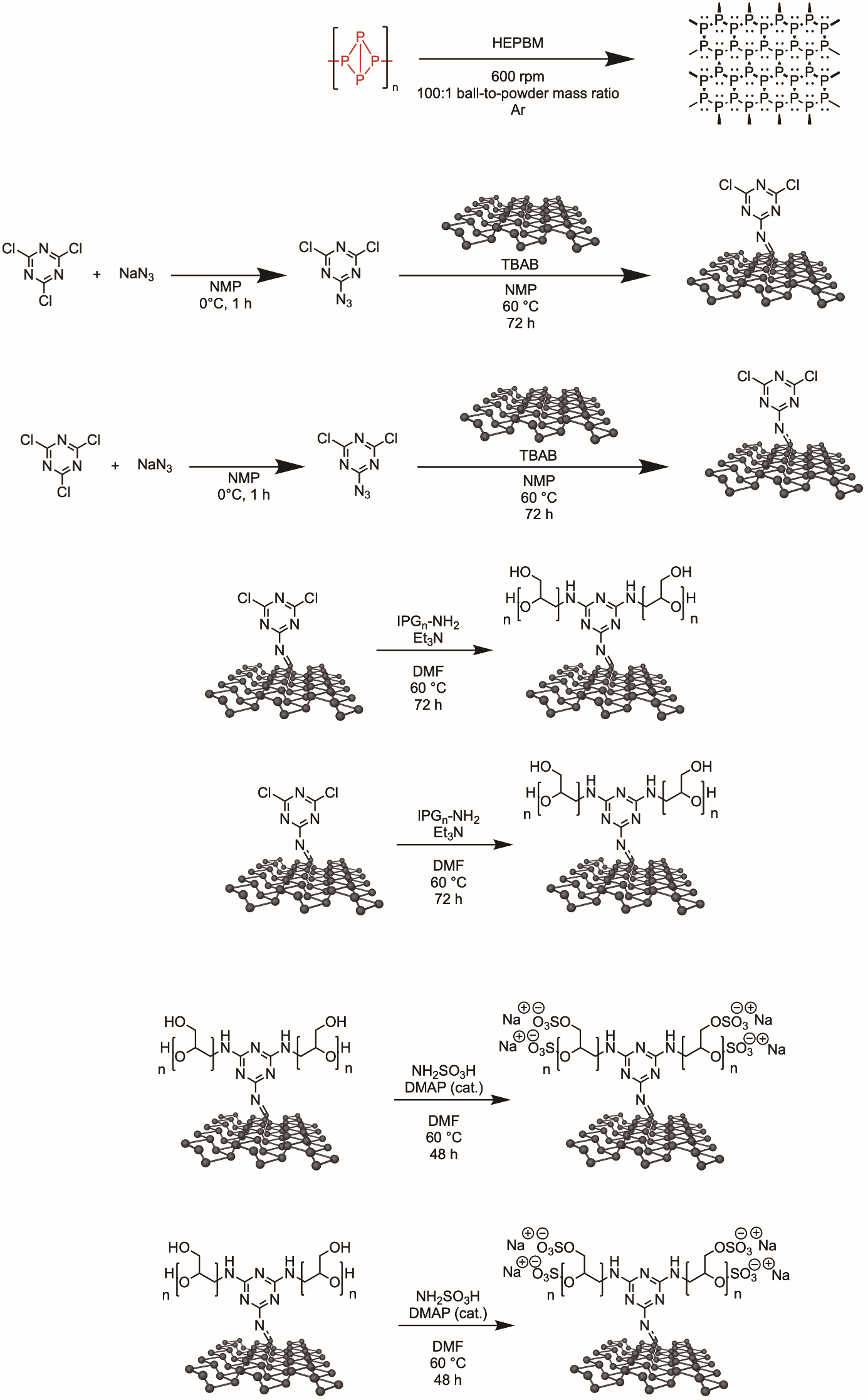
**

**Scheme S2.** Synthetic pathway of BPNS-Trz.

Cyanuric chloride (2.40 g, 13.0 mmol, 1.0 eq.) was dissolved in dry NMP (20 mL) in a 100 mL schlenk flask and cooled to 0 °C in an ice bath. Sodium azide (0.84 g, 12.9 mmol, 1.0 eq.) was added slowly upon stirring in an argon counterflow. The reaction mixture was stirred for at 0°C for 1 h to form cyanuric azide. In parallel, exfoliated BPNS (200 mg) were redispersed in NMP (40 mL) and a catalytic amount of TBAB was added (~2 mg). The dispersion was purged with nitrogen for 20 min and added to the cyanuric azide solution. The reaction mixture was stirred for 72 h at 60 °C. After cooling to room temperature, the product was isolated by centrifugation (20 min, 9500 rpm, 4 °C). Further, the residue was washed by six repeated washing steps using a 1:1 mixture of acetone and deionized water (50 mL). The product was obtained as black powder after lyophilization (150 mg) and stored under argon.

### Synthesis of 2-azido-4,6-dichloro-1,3,5-triazine

**Scheme S3.** Scheme for the synthesis of 2-azido-4,6-dichloro-1,3,5-triazine.

Following a protocol from Sharma et al.^[2]^ 2-azido-4,6-dichloro-1,3,5-triazine was synthesized. Briefly, cyanuric chloride (5.0 g, 27.3 mmol, 1.0 eq.) was dissolved in acetone (100 mL) and cooled to 0°C in an ice bath. In parallel, sodium azide (1.77 g, 27.3 mmol, 1.0 eq.) was dissolved in water (50 mL) and cooled to 0°C. The aqueous sodium azide solution was added dropwise to the cyanuric chloride solution under vigorous stirring while maintaining 0°C. The mixture was stirred at this temperature for until completion (30 min), before removing the acetone under reduced pressure at ice cold conditions. The remaining aqueous phase was extracted with cold dichloromethane (3 x 50 mL), the combined organic layers were dried over MgSO_4_ and filtered. The solvent was removed under reduced pressure at ice cold conditions. The crude product was purified using column chromatography (*n*-hexane). The product (4.3 g, 83%) was obtained as a colorless solid.

### Synthesis of 3,5-dichloro-*N-*(triphenylphosphoranylidene)aniline

**Scheme S4.** Scheme for the synthesis of 3,5-dichloro-*N-*(triphenylphosphoranylidene)aniline.

Following a modified protocol from Meguro et al.^[3]^ 3,5-dichloro-*N-*(triphenylphosphoranylidene)aniline was synthesized. In a 10 mL schlenk-flask 1-azido-3,5-dichlorobenzene (0.05 g, 0.31 mmol, 1.0 eq.) was dissolved in dry DMF (2 mL). Triphenylphosphine (0.097 g, 0.37 mmol, 1.2 eq.) was added in an argon counter flow under stirring. The reaction mixture was stirred at room temperature for 24 h. After completion, the solvent was removed under reduced pressure. The crude product was purified using column chromatography (*n*-pentane/EtOAc = 5/1) to give the product (0.05 g, 38%) as a colorless solid.

**^1^H-NMR** (600 MHz, CDCl_3_): *δ* [ppm] = 7.75-7.69 (m, 6H), 7.58-7.54 (m, 3H), 7.50-7.46 (m, 6H), 6.62-6.60 (m, 3H).

**^31^P-NMR** (600 MHz, CDCl_3_): *δ* [ppm] = 5.72 (m).

**MS** (ESI): m/z = 422.06 [M]^+·^.

### Synthesis of *N-*(4,6-dichloro-1,3,5-triazine)triphenylphosphoran-ylidene

**Scheme S5.** Scheme for the synthesis of *N-*(4,6-dichloro-1,3,5-triazine)triphenylphosphoranylidene.

Following a modified protocol from Meguro et al.^[3]^ *N-*(4,6-dichloro-1,3,5-triazine)triphenylphosphoranylidene was synthesized. In a 10 mL schlenk-flask 2-azido-4,6-dichloro-1,3,5-triazine (0.10 g, 0.52 mmol, 1.0 eq.) was dissolved in dry DMF (3 mL). Triphenylphosphine (0.165 g, 0.63 mmol, 1.2 eq.) was added in an argon counter flow under stirring. The reaction mixture was stirred at room temperature for 24 h. After completion, the solvent was removed under reduced pressure. The crude product was purified using column chromatography (*n*-pentane/EtOAc = 5/1) to give the product (0.12 g, 54%) as a colorless solid.

**^1^H-NMR** (600 MHz, CDCl_3_): *δ* [ppm] = 7.84-7.78 (m, 2H), 7.63-7.58 (m, 1H), 7.52-7.48 (m, 2H).

**^31^P-NMR** (600 MHz, CDCl_3_): *δ* [ppm] = 22.48 (m).

**MS** (ESI): m/z = 447.03 [M-Na]^+^, 463.00 [M-K]^+^.

### Synthesis of N-(diphenylphosphaneyl)-N-1,1-triphenylphosphanamine

**Scheme S6.** Scheme for the synthesis of N-(diphenylphosphaneyl)-N-1,1-triphenylphosphanamine.

Following a protocol from Eady et al.^[4]^ N-(diphenylphosphaneyl)-N-1,1-triphenylphosphanamine was synthesized. In a 10 mL schlenk-flask chlorodiphenylphosphine (1.08 mL, 2.0 mmol, 2 eq.) was added dropwise to a solution of aniline (0.09 mL, 1.0 mmol, 1 eq.) and triethylamine (1.4 mL, 10 mmol, 10 eq.) in DCM (5 mL) while stirring. The formation of a white precipitated could be observed. The reaction mixture was stirred at room temperature for 12 h. After completion, the solvent was removed under reduced pressure and the solid was thoroughly washed with MeOH (5 x 20 mL). The crude product was recrystallized from dichloromethane/hexane (1:1) at room temperature. The product (0.036 g, 78%) was obtained as a colorless solid.

**^1^H-NMR** (600 MHz, CDCl_3_): *δ* [ppm] = 7.38-7.29 (m, 20H), 6.98-6.92 (m, 3H), 6.67-6.64 (m, 2H).

**^31^P-NMR** (600 MHz, CDCl_3_): *δ* [ppm] = 69.12 (s).

**MS** (ESI): m/z = 462.15 [M-H]^+^.

### Synthesis of BPNS-triazine linear-polyglycerol (BPNS-Trz-lPG_n_)

**
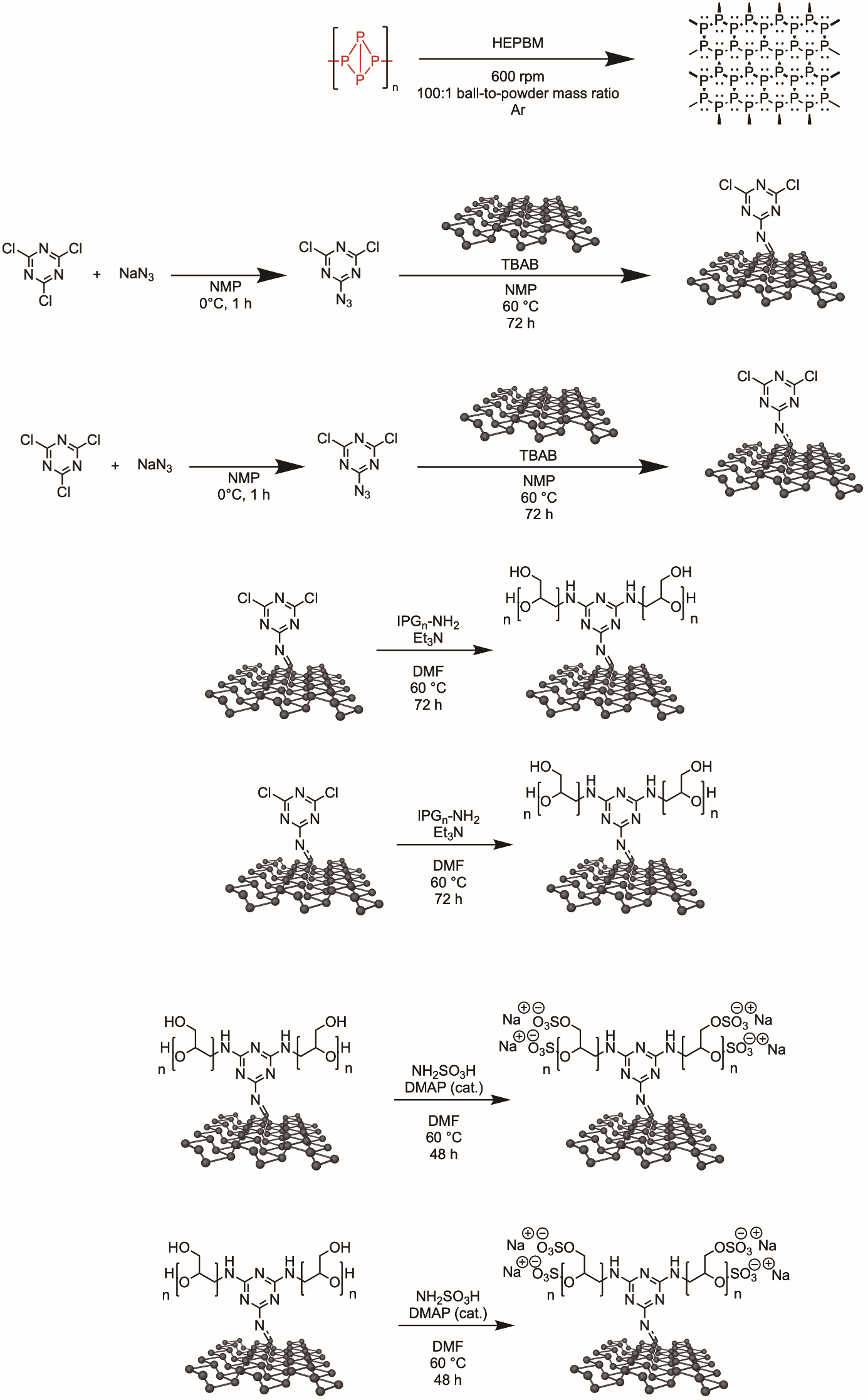
**

**Scheme S7.** Reaction conditions for the synthesis of BPNS-Trz-lPG_n_.

In a schlenk flask, linear polyglycerol (1 eq.) was dissolved in dry DMF to a final polymer concentration of 15 mg mL^-1^ and cooled to 0 °C in an ice bath. Next, triethyl amine (TEA (19 eq.)) was added upon stirring. BPNS-Trz was dispersed in dry DMF (3 mg mL^-1^, using a volume equal to the polymer solution) and sonicated for 30 min before being added to the reaction flask. The reaction flask was heated to 60 °C and stirred for 72 h. The reaction mixture was cooled to room temperature and centrifuged (5 min, 5000 rpm, 4 °C). The supernatant was collected and dialyzed (100 kDa MWCO) against H_2_O for 3 d. The aqueous solution was lyophilized and the product was obtained as a brown viscous solid (BPNS-Trz-lPG_7_: 87%, BPNS-Trz-lPG_30_: 96%) and stored under argon.

| BPNS-Trz-lPG_7_: | **^1^H-NMR** (500 MHz, D_2_O): δ [ppm]: 3.86-3.55 (m, lPG-groups) |
| --- | --- |
|  | **^13^C-NMR** (125 MHz, D_2_O): δ [ppm]: 80-60 (lPG-goups) |
| BPNS-Trz-lPG_30_: | **^1^H-NMR** (500 MHz, D_2_O): δ [ppm]: 3.88-3.61 (m, lPG-groups) |
|  | **^13^C-NMR** (125 MHz, D_2_O): δ [ppm]: 80-60 (lPG-goups) |

### Synthesis of BPNS-triazine linear-polyglycerol-sulfate (BPNS-Trz-lPGS_n_)

**
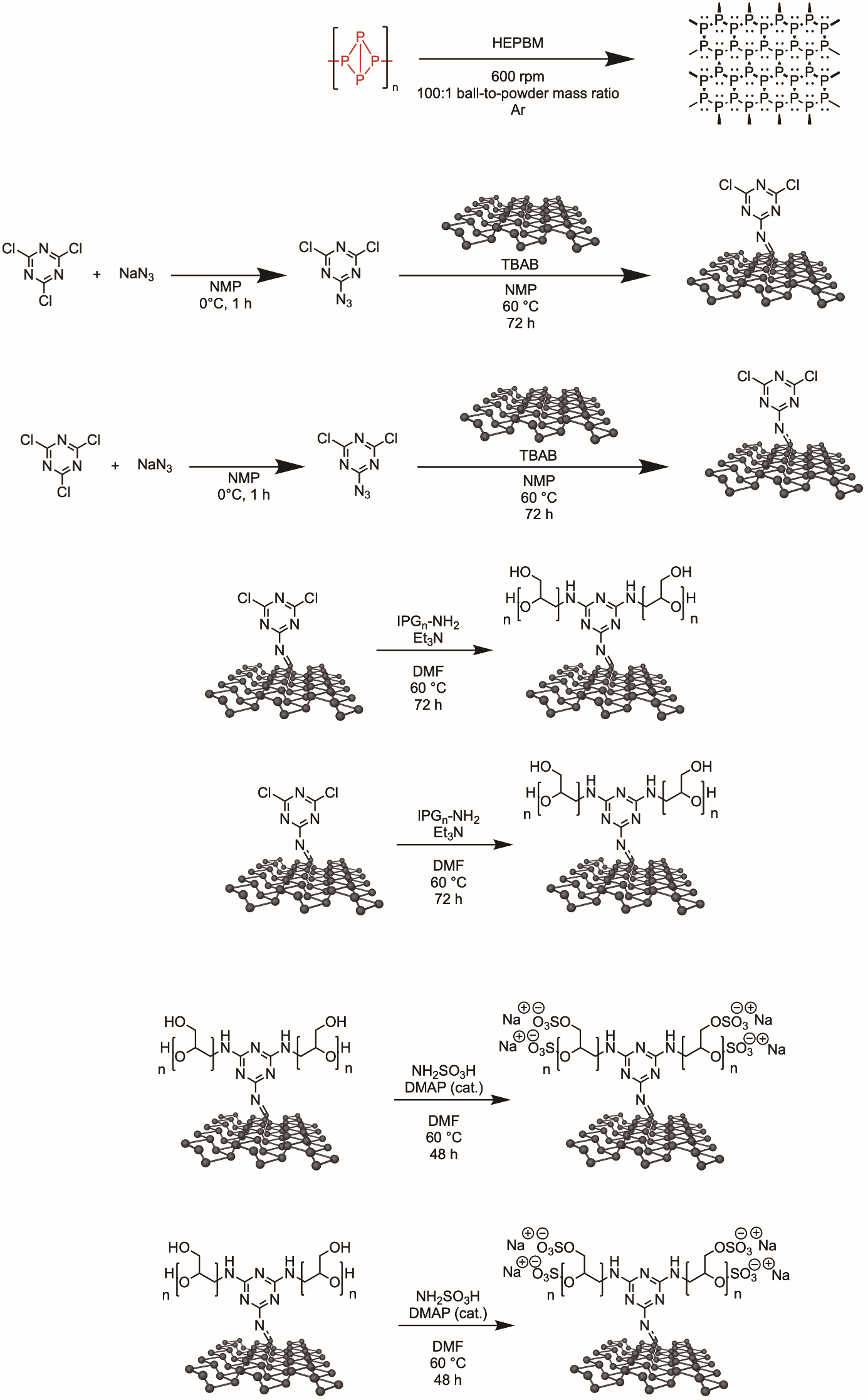
**

**Scheme S8.** Reaction conditions for the sulfation of BPNS-Trz-lPG_n_.

In a schlenk flask, BP-Trz-lPG_n_ (1 eq.) was dissolved in dry DMF to a final polymer concentration of 5 mg mL^-1^. Sulfamic acid (1.5 eq./OH group) and DMAP (cat.) were added upon stirring. The reaction flask was heated to 60 °C and stirred for 48 h. The reaction mixture was set to a basic pH (> 10) using NaOH and was dialyzed (2 kDa MWCO) against 1.0 M NaCl-solution (pH 10) for 2 d, followed by deionized water until the solution was neutral. The aqueous solution was lyophilized and the product was obtained as a brown solid and stored under argon.

| BPNS-Trz-lPG_7_: | **^1^H-NMR** (500 MHz, D_2_O): δ [ppm]: 4.31-3.71 (m, lPG-groups) |
| --- | --- |
| BPNS-Trz-lPG_30_: | **^1^H-NMR** (500 MHz, D_2_O): δ [ppm]: 4.28-3.65 (m, lPG-groups) |

# Supplementary figures and tables

**Table S2**. Characterization of BP derivatives starting from pristine BP, with elemental analysis and zeta potential values taken at 1.0 mg mL^-1^.

| Compound | ζ  [mV] | C  [%] | H  [%] | N  [%] | S  [%] |
| --- | --- | --- | --- | --- | --- |
| BPNS | -30.7 | 2.7 | 2.0 | 0.0 | 0.0 |
| BPNS-Trz | -33.6 | 17.7 | 4.5 | 5.7 | 0.0 |
| lPG_7_-NH_2_ | - 22.7 | 47.5 | 7.5 | 0.2 | 0.0 |
| lPG_30_-NH_2_ | - 26.2 | 47.8 | 7.6 | 0.1 | 0.0 |
| BP-Trz-lPG_7_ | - 15.4 | 57.1 | 6.0 | 0.4 | 0.0 |
| BP-Trz-lPG_30_ | - 16.7 | 46.3 | 10.4 | 0.2 | 0.0 |
| BP-Trz-lPGS_7_ | - 57.5 | 19.1 | 6.8 | 0.2 | 17.0 |
| BP-Trz-lPGS_30_ | - 51.1 | 22.2 | 4.2 | 0.2 | 11.1 |

**
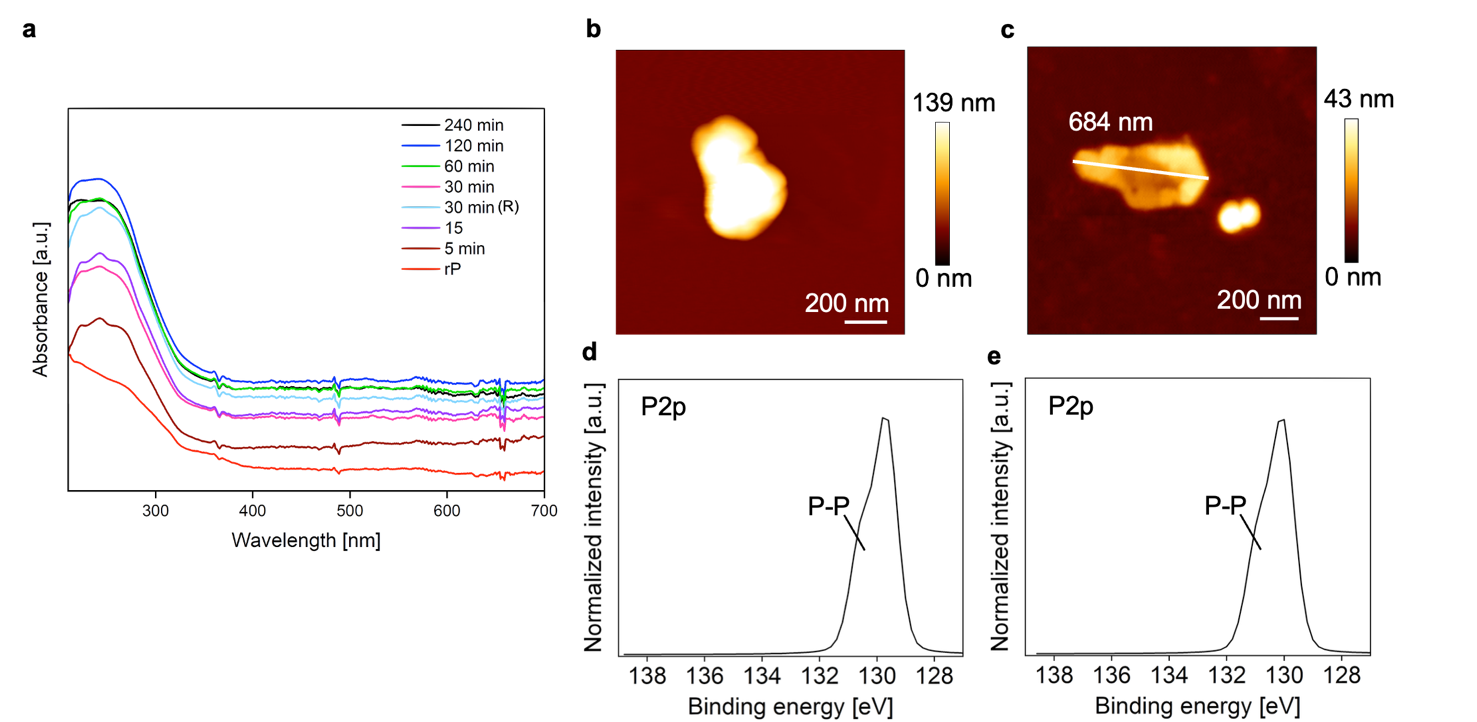
**

**Figure S1.** (a) UV/Vis adsorption of BP at different milling times. Representative AFM images of (b) BPNS exfoliated in NMP via bath sonication (the scale bar corresponds to 200 nm) and (c) BPNS exfoliated in NMP via probe sonication (the scale bar corresponds to 200 nm). Highly resolved P2p XP spectra with peak fitting for (d) BPNS exfoliated in NMP via bath sonication and (e) BPNS exfoliated in NMP via probe sonication.

BPNS production from red phosphorus (RP) was optimized by a planetary ball-milling approach. The conversion of RP to BP (Figure 1a) was monitored using Raman spectroscopy, XRD and UV/Vis spectroscopy (Figure 1b, S1). Compared to RP, BP had three characteristic peaks in Raman spectroscopy at ~360 cm^-1^, ~430 cm^-1^ and ~460 cm^-1^ corresponding to the $A_{g}^{1}$, $B_{2g}$ and $A_{g}^{2}$ vibrational modes respectively (Figure 1b). XRD patterns revealed the appearance of the crystalline BP reflections ((020), (021), (040), and (111)) after 30 min of milling in reverse mode (30 (15 + 15) min R) (Figure 1b). UV/Vis further indicated complete conversion from RP to BP after 30 min of milling with the reverse mode (30 min R) (Figure S1). The high-resolution P2p XP spectrum of pristine RP indicated partial oxidation evidenced by the P-O peak at 134.9 eV (Figure 1c). Notably, converted BP showed no sign of oxidation, which suggests reductive conditions during the ball-milling process (Figure 1d). Next, BPNS were prepared via liquid-phase exfoliation in *N*-methyl-2-pyrrolidone (NMP) using sonication. Bath sonication produced BP nanosheets with comparable dimensions (Figure S1) to those obtained by probe sonication but with a lower yield (~15 mg from 1 g of BP). The probe method markedly improved exfoliation efficiency and overall yield of BPNS (~150 mg from 1 g of BP). The resulting BPNS exhibited an average thickness of ~50 nm with lateral sizes of ~1-2 µm (Figure S1). The high-resolution P2p spectra for both methods showed no sign of oxidation (Figure S1) and the absence of nitrogen signals in the survey spectrum confirmed efficient removal of NMP (Figure S2).

**
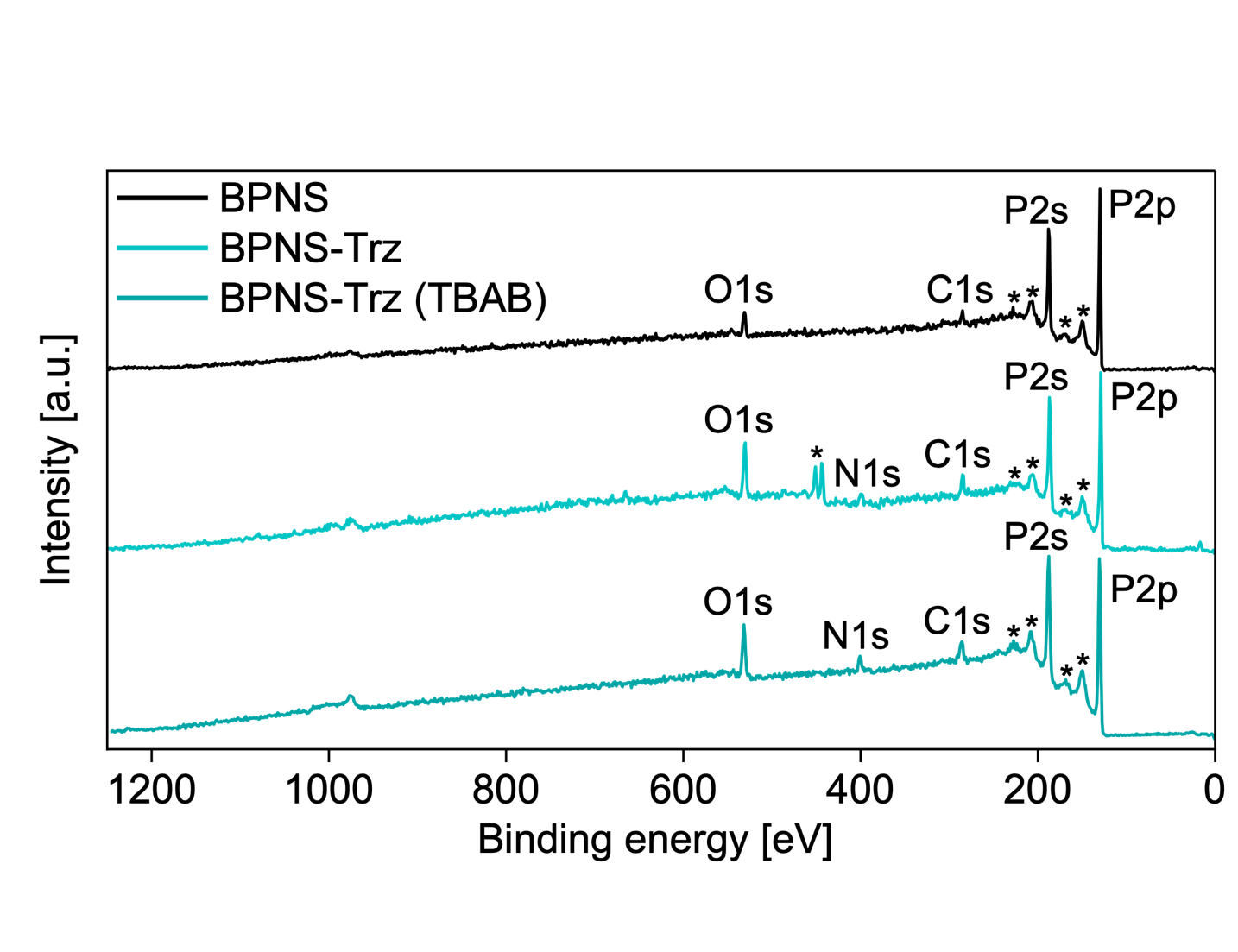
**

**Figure S2.** XP survey spectra for BPNS and BPNS-Trz with and without the catalyst (peaks marked with * correspond to signals from the indium substrate background).

**Table S3.** Relative elemental fractions from the quantification of the XPS survey of BPNS and BPNS-Trz (TBAB).

|  | BE | BPNS  at% | BPNS-Trz  at% |
| --- | --- | --- | --- |
| O1s | 530 eV | 11 | 18 |
| N1s | 400 eV | 0 | 7 |
| C1s | 285 eV | 7 | 12 |
| P2s | 187 eV | 38 | 28 |
| P2p | 130 eV | 46 | 37 |

**
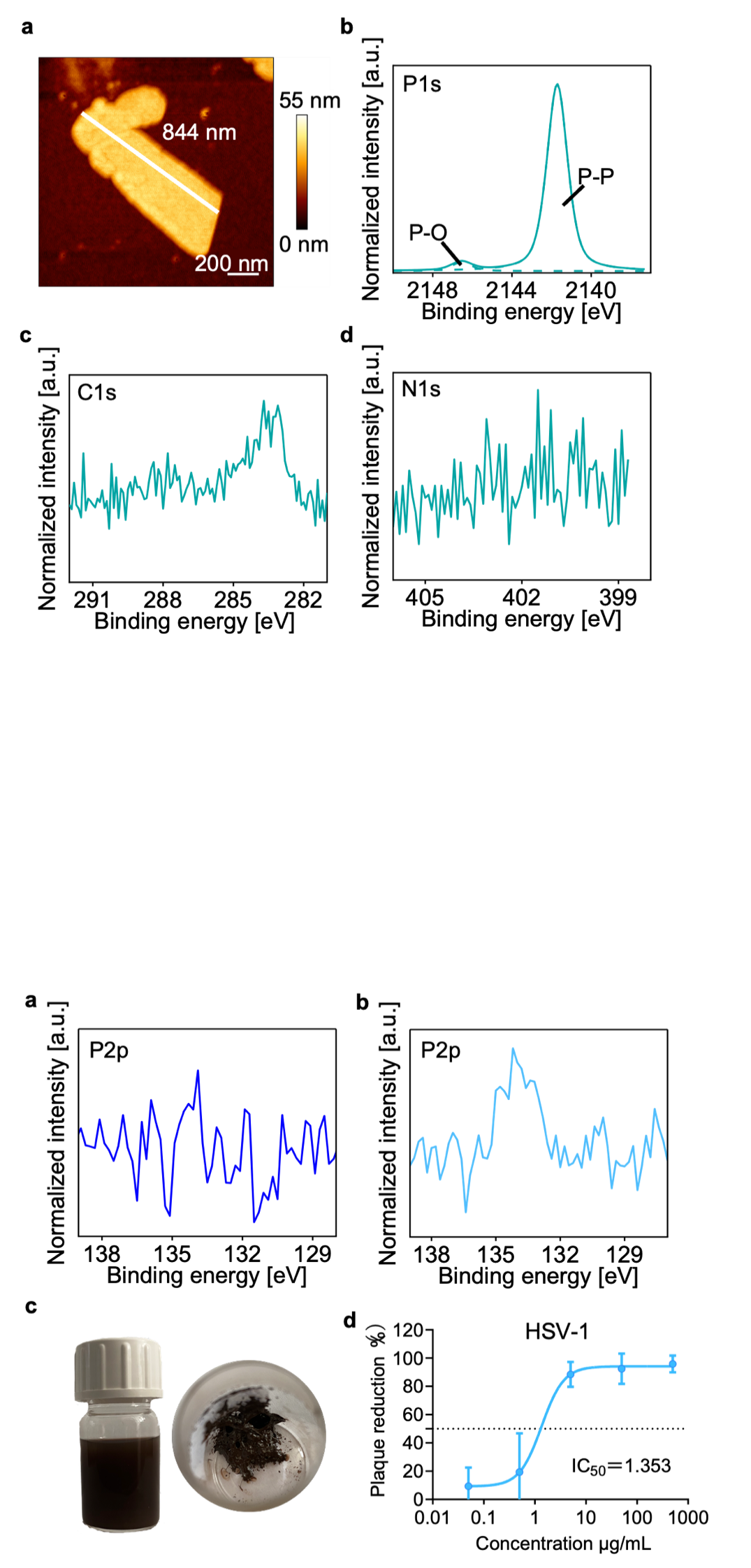
**

**Figure S3.** (a) Representative AFM image of BPNS-Trz. High resolution HAXPES spectra with peak fitting for BPNS-Trz (b) P1s, (c) C1s and (d) N1s.

XPS analysis provided direct evidence for covalent conjugation of triazine moieties to BPNS moiety and allowed quantification of the functionalization degree. A N1s signal emerged in the survey spectrum of BPNS-Trz, that was absent in unfunctionalized BPNS (Figure S2). To further increase the functionalization degree, tetrabutylammonium bromide (TBAB) was used as a phase-transfer catalyst. It resulted in a significant N1s component increase from ~3% to ~7%, that corresponds to one triazine group per ~50 phosphorus atoms (Table S3). Notably, highly similar C1s and N1s XP spectra of BPNS-Trz (with and without catalyst) gave highly reproducible results and confirmed the presence of triazine moieties on the surface of BPNS (Figure 2c,d,g,h). In the high resolution C1s XP spectra of BPNS-Trz with and without the catalyst three components at 285.5 eV, 287.0 eV and 289.2 eV corresponded to the C-N=C, C-Cl and C-N-P bonds of the dichlorotriazine groups, respectively (Figure 1d,h). The high-resolution N1s XP spectra exhibited two components attributed to the N-P bonds between BPNS and triazine as well as N-C=N bonds within the triazine ring (Figure 2c,g). The relative peak areas of 1:3 aligned with the stoichiometric distribution of three nitrogen atoms in the triazine ring and the single nitrogen atom covalently bonded to the BPNS. In the high-resolution P2p XP spectra, for both materials three peaks were observed. P-P bond at 130.1 eV, P=N bond at 133.4 eV and some oxidized P-O_x_ species at 135.0 eV respectively (Figure 1b,f). The high-resolution P2p XP spectra offered a clear difference in the degree of functionalization of BPNS. For the sample prepared without the catalyst, the intensity of P=N component was significantly lower compared with the sample prepared with the use of phase-transfer catalyst (Figure 1i).

Additionally, HAXPES was used for probing greater sample depth of the overall bonding environment. In the high resolution P1s spectrum a signal for P-P (2142.5 eV) and P-O (2146.2 eV) were observed (Figure S4). Owing to the increased information depth of HAXPES underlying BPNS were detectable. In the high-resolution HAXPES C1s and N1s spectra, no signals were observed (Figure S4). These findings confirmed that the triazine moiety formed a covalent bond to the BPNS surface. AFM revealed a surface morphology with heights up to 55 nm and similar lateral dimensions as BPNS (Figure S4). Next, ToF-SIMS analysis confirmed the presence and spatial distribution of triazine-derived fragments. Both BPNS and BPNS-Trz exhibited a fragment corresponding to PO_2_^-^ on the surface (Figure 2m,o). In contrast, a CN⁻ fragment, that is characteristic to the triazine moiety, was detected exclusively for BPNS-Trz (Figure 2n,p). The CN⁻ signal was homogeneously distributed across the analyzed area, indicating uniform surface functionalization after the covalent conjugation of triazine moieties. SEM-EDS mapping further supported revealed homogeneous surface coverage and uniform elemental distribution in the BPNS-Trz (Figure 2q). Fourier transform infrared (FTIR) spectroscopy of 2-azido-4,6-dichloro-1,3,5-triazine exhibited a characteristic -N_3_ stretching vibration at 2169 cm^-1^. Pristine BPNS displayed bands at 1201 cm^­­-1^ (P=O), 993 cm^-1^, and 900 cm^-1^ (P-O), that arise from minor surface oxidation during sample handling under ambient conditions (Figure 2i). After reaction with the 2-azido-4,6-dichloro-1,3,5-triazine, a new absorption band at 1013 cm^­‑1^ appeared, that corresponds to P-N bond formation.^[5]^ Raman spectra of BPNS-Trz retained the three characteristic BP modes ($A_{g}^{1}$, $B_{2g}$ and $A_{g}^{2}$). A slight red shift was observed (Figure 2j), consistent with hindered lattice vibrations of covalently bound phosphorus atoms. This trend agrees with previous reports on covalently functionalized BP.^[6]^ To further confirm the successful functionalization and to elucidate the binding environment NEXAFS studies were performed at the carbon and nitrogen K-edges. NEXAFS spectra at the C and N K-edges were consistent with previous studies on dichlorotriazine-functionalized graphene.^[7]^ The NEXAFS C K-edge spectrum of BPNS-Trz revealed several resonance features related to the dichlorotriazine moiety (Figure 1k). A pronounced resonance at 289.5 eV was attributed to the C1s π* transition of the sp^2^-hybridized C=N bonds. Another sharp resonance feature was observed at slightly higher binding energies (290.3 eV) referred to the C1s σ* transition of C-N bonds. In the higher energy region between 292.6 eV and 296.7 eV various overlapping resonance features aroused as the C1s σ* transitions of the dichlorotriazine moiety. The most significant feature at 295.5 eV was associated with the C1s σ* (C-Cl) transition (Figure 1k). At the N K-edge, the NEXAFS spectrum exhibited two main peaks at 399.0 eV and 400.8 eV assigned to N1s π* (N=C) and (N=C-Cl) resonances respectively, corresponding to the aromatic nitrogen species within the dichlorotriazine moiety (Figure 2l). In addition, another resonance feature at 401.2 eV attributed to the N1s σ* (N-P) transition, confirms the covalent functionalization of the BPNS.

**
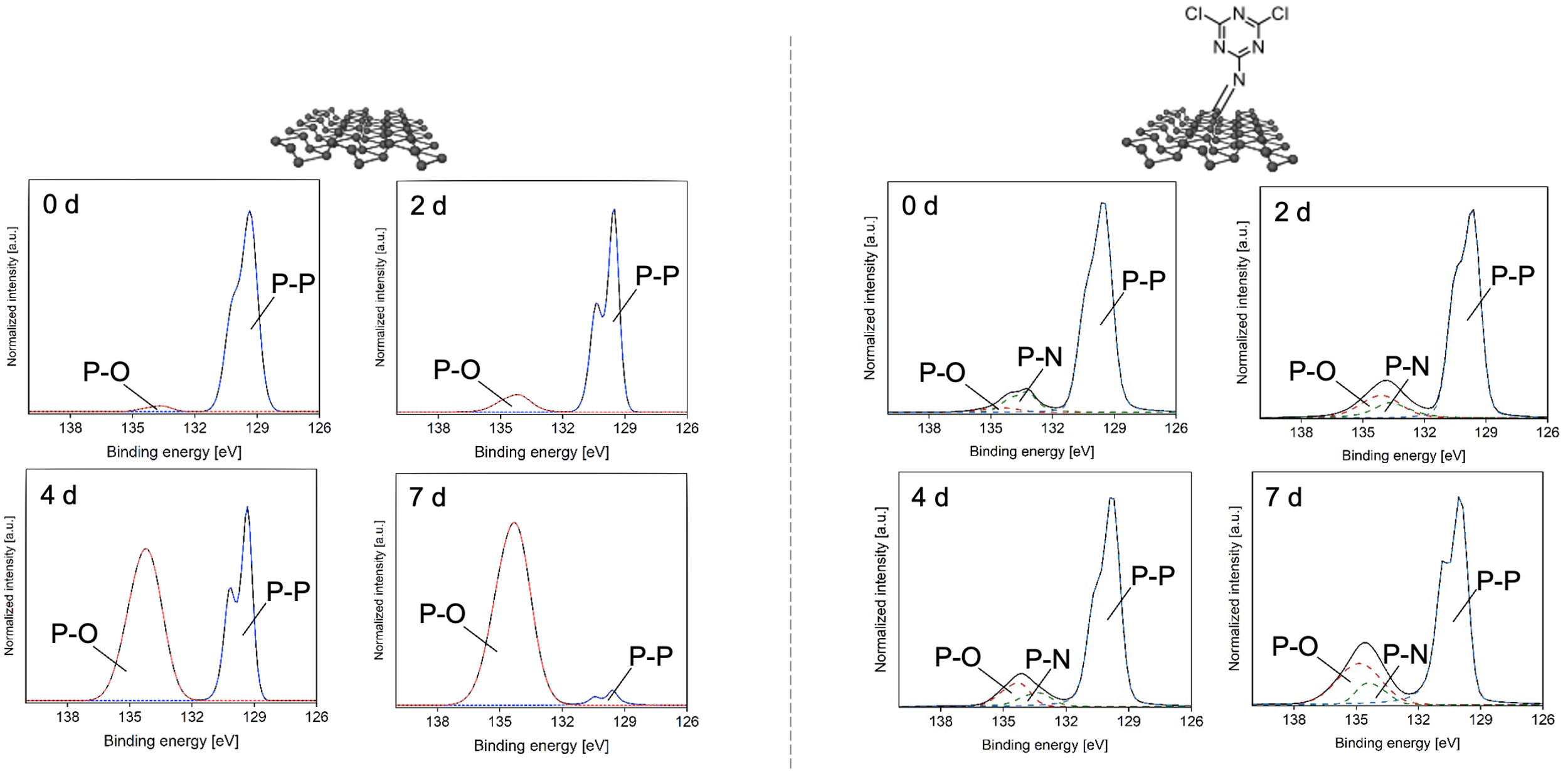
**

#### **Figure S4.** Stability experiments for the degradation of BP and BPNS-Trz. Highly resolved XPS P2p spectra for BP (left) and BPNS-Trz (right) after 0d, 2d, 4d and 7d.

The stability of BP and the exfoliated BPNS-Trz were systematically investigated by XPS (Figure S3). The samples were stored under ambient conditions and high-resolution P2p spectra were recorded daily. BP displayed rapid oxidation after 2 d, increasing to ~50% after 4 d and nearly complete surface oxidation after 7 d (Figure S3). In contrast, BPNS-Trz revealed remarkable stability, with slight oxidation detected after 7d of exposure to ambient conditions.


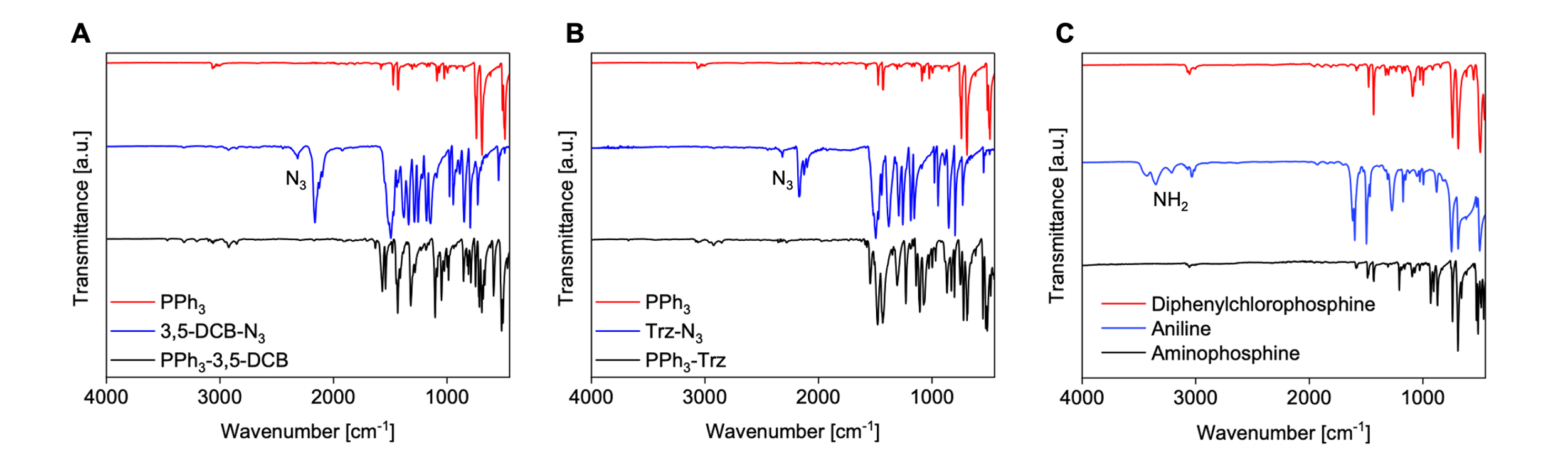


**Figure S5.** IR comparison of synthesized P=N/P-N control materials. Disappearance of the N_3_ and NH_2_ signal correspond to successful conversion.


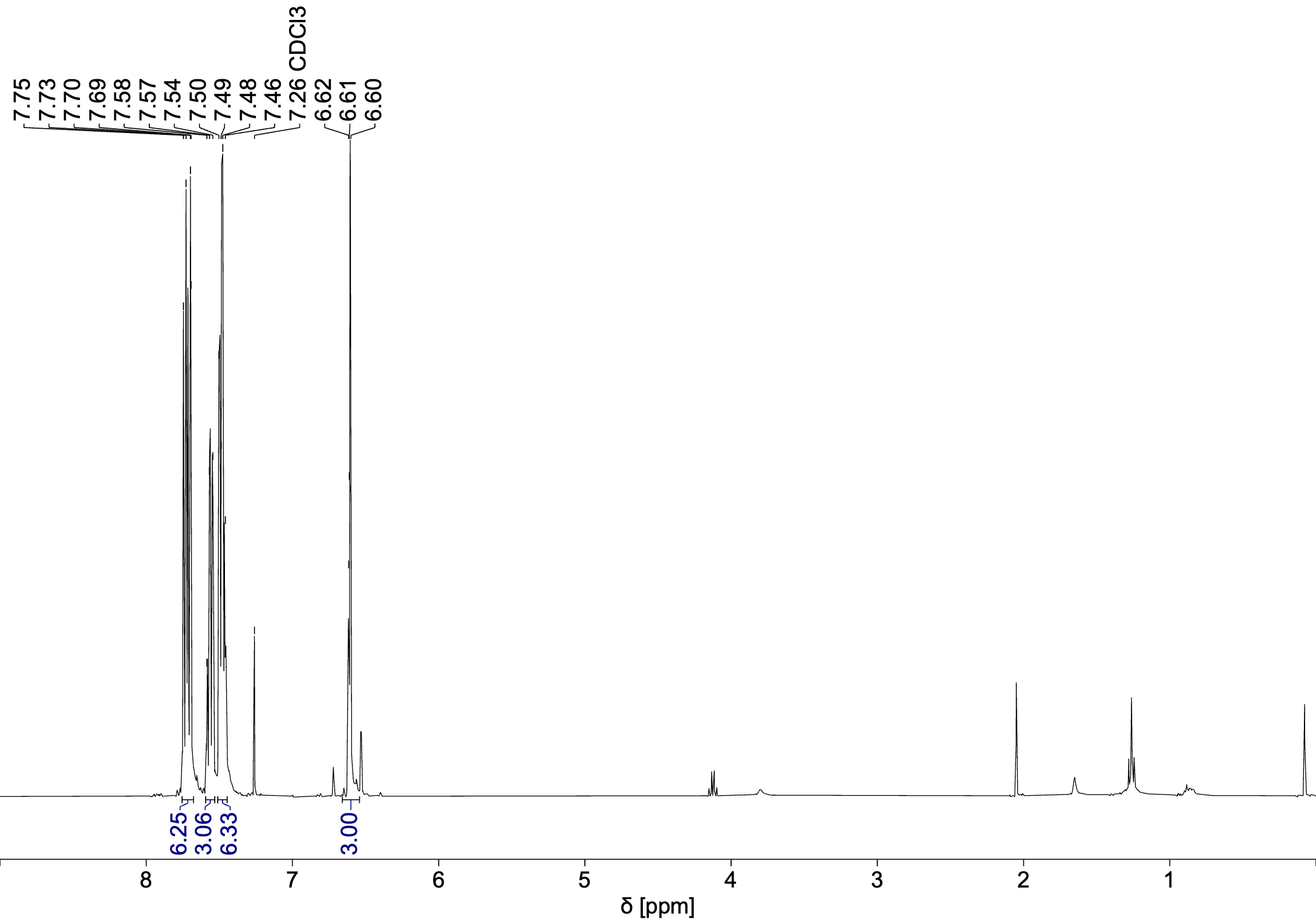


#### **Figure S6.** ^1^H-NMR of 3,5-dichloro-*N-*(triphenylphosphoranylidene)aniline.


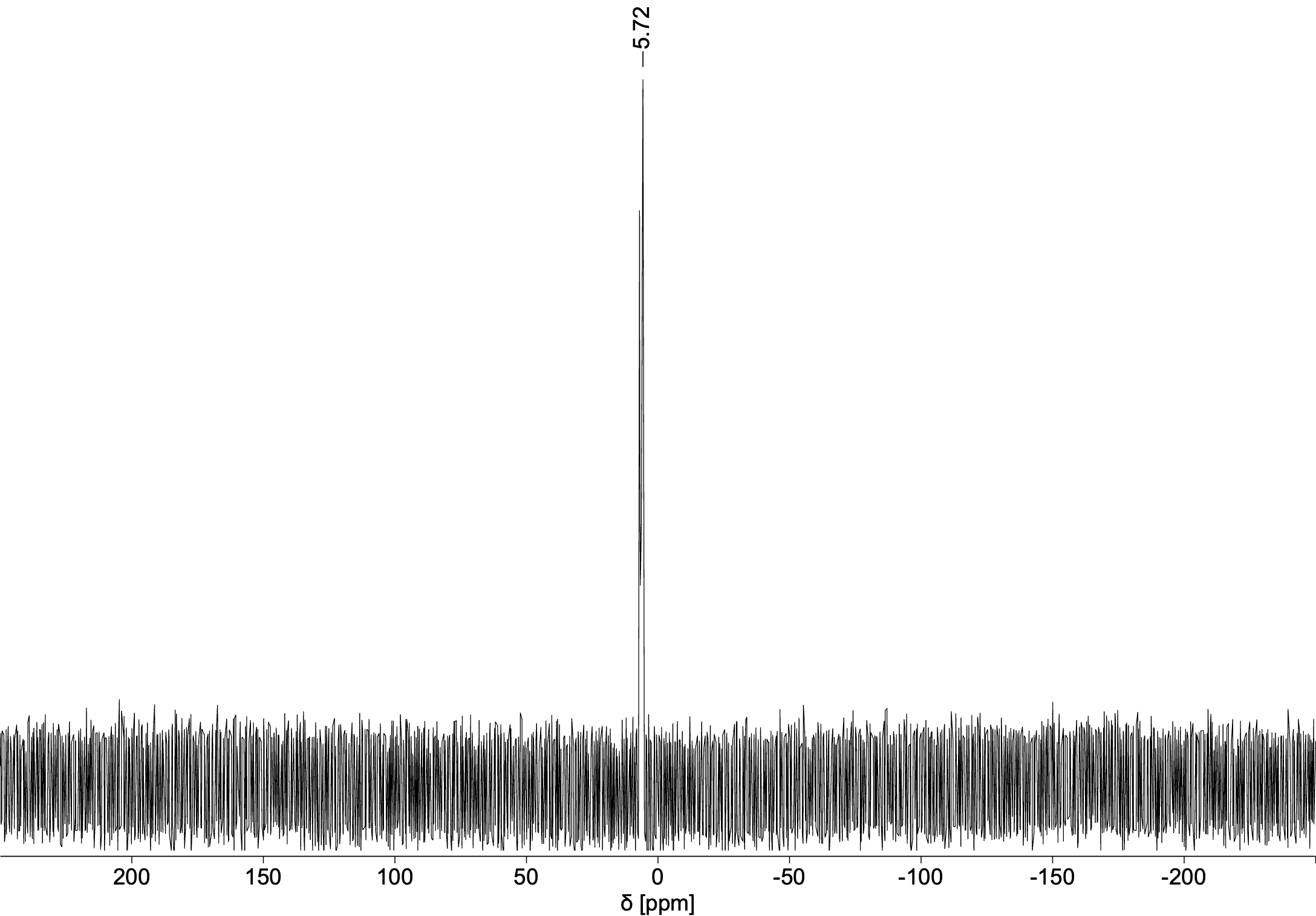


#### **Figure S7.** ^31^P-NMR of 3,5-dichloro-*N-*(triphenylphosphoranylidene)aniline.

#### **Figure S8.** ESI of 3,5-dichloro-*N-*(triphenylphosphoranylidene)aniline.


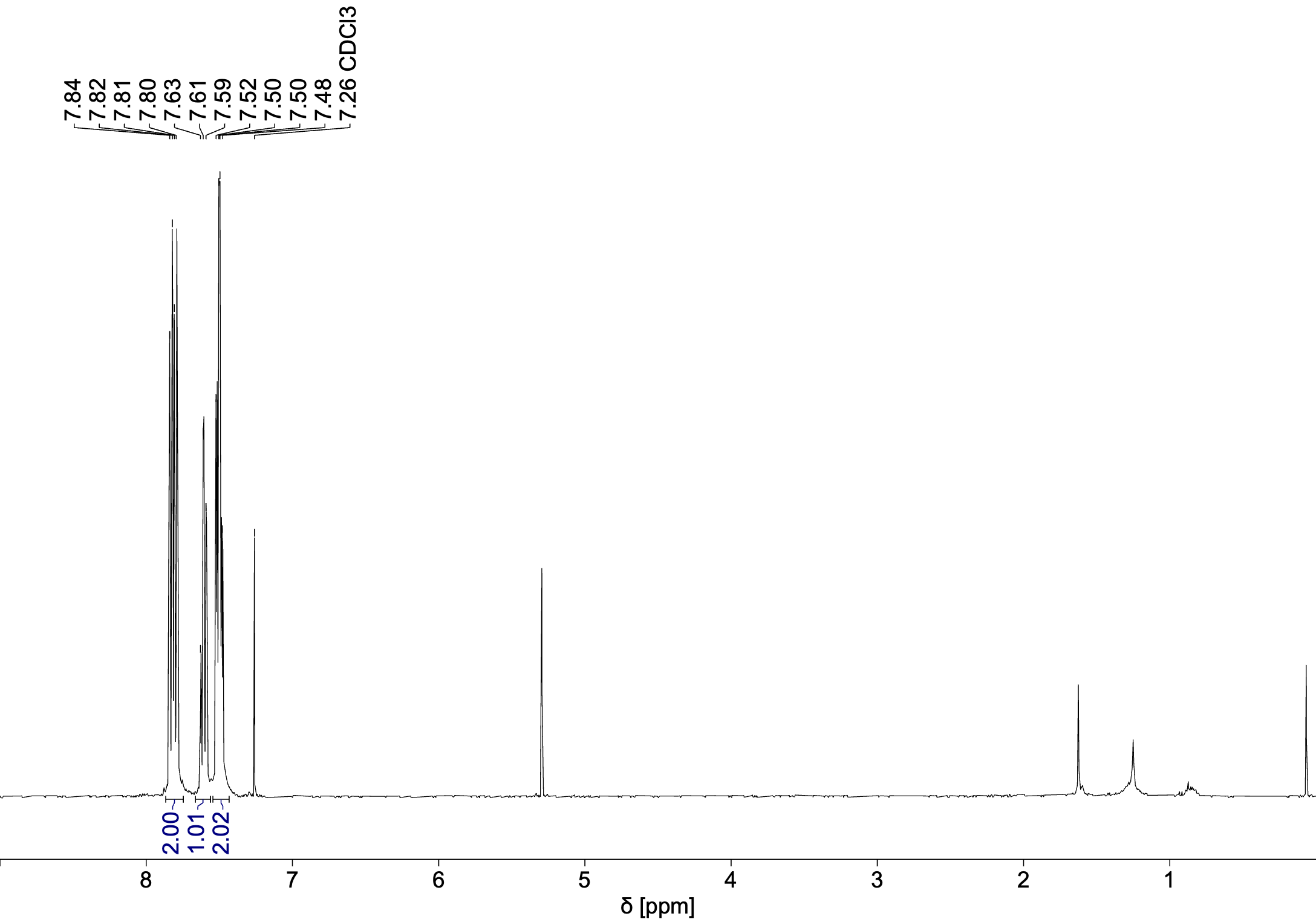


#### **Figure S9.** ^1^H-NMR of *N-*(4,6-dichloro-1,3,5-triazine)triphenylphosphoranylidene.


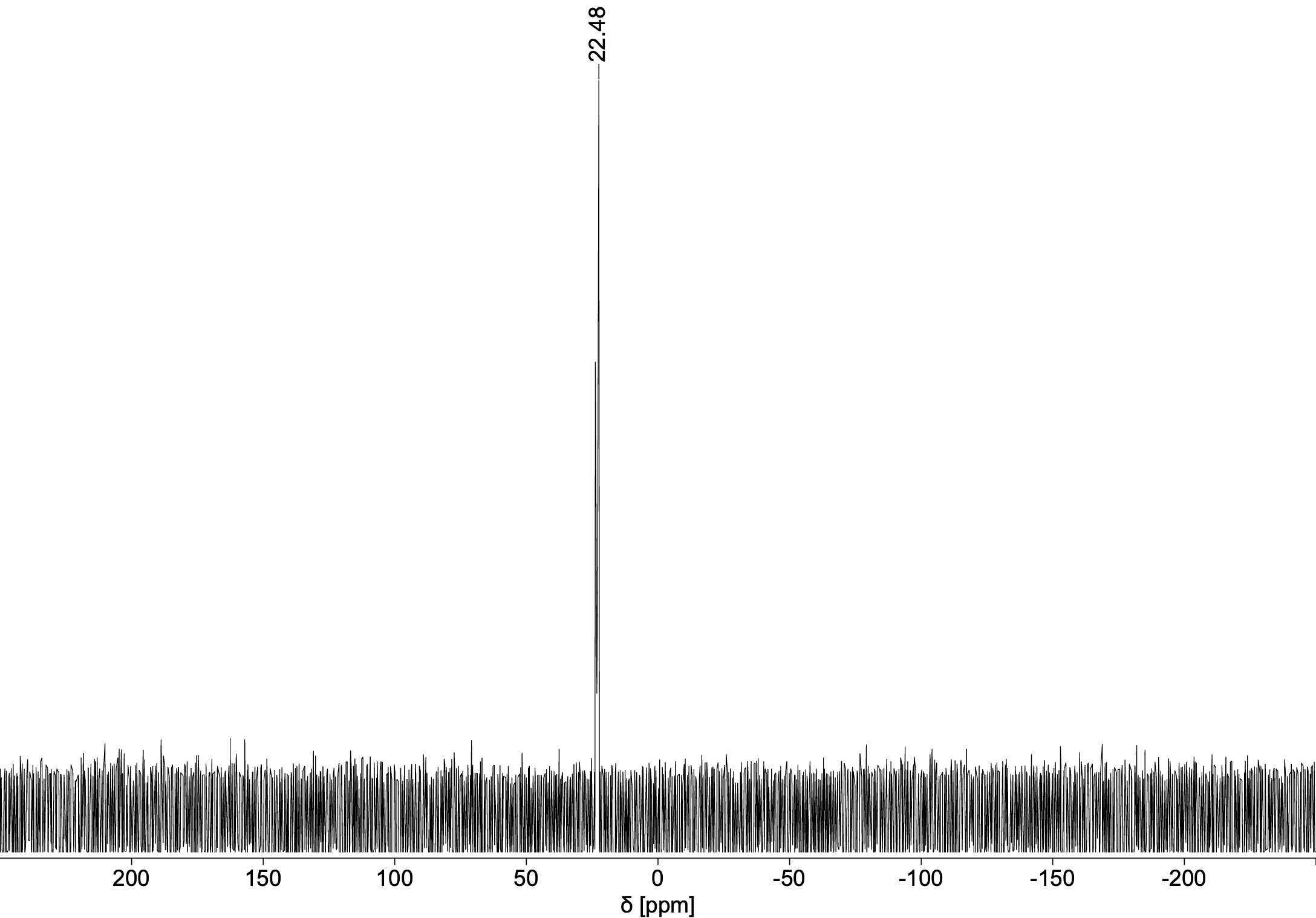


#### **Figure S10.** ^31^P-NMR of *N-*(4,6-dichloro-1,3,5-triazine)triphenylphosphoranylidene.

#### **Figure S11.** ESI of *N-*(4,6-dichloro-1,3,5-triazine)triphenylphosphoranylidene.


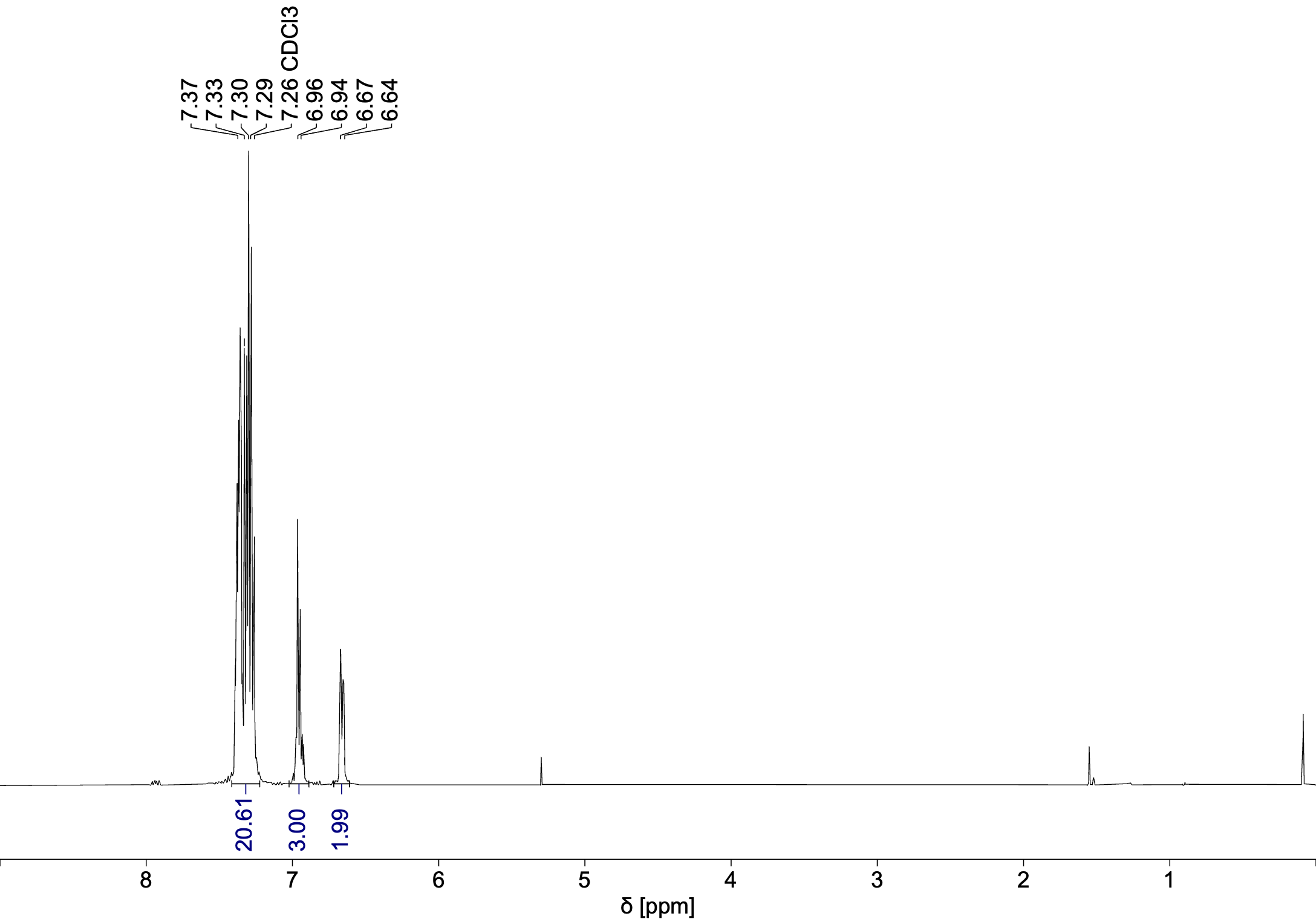


#### **Figure S12.** ^1^H-NMR of *N*-(diphenylphosphaneyl)-*N*-1,1-triphenylphosphanamine.


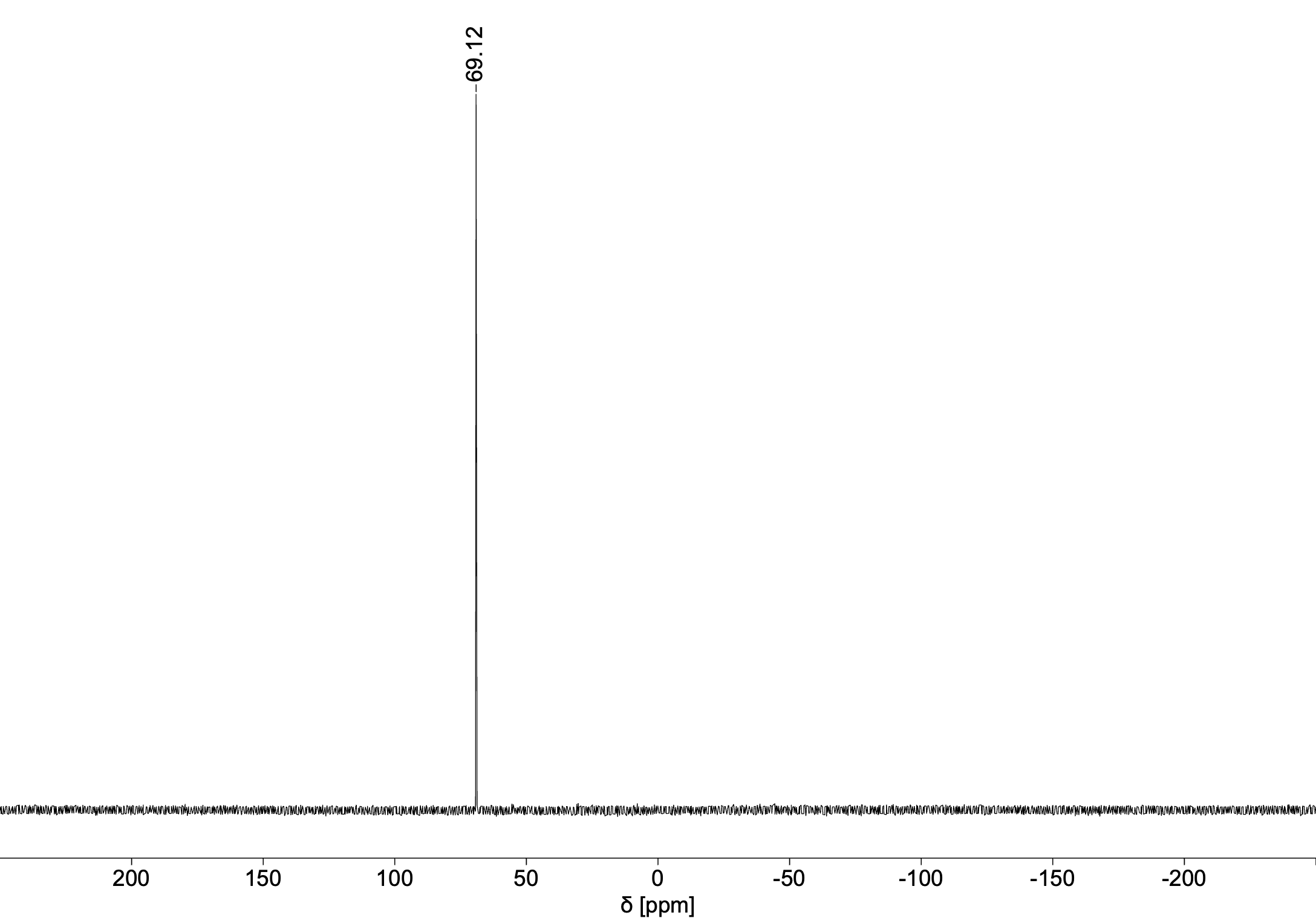


#### **Figure S13.** ^31^P-NMR of *N*-(diphenylphosphaneyl)-*N*-1,1-triphenylphosphanamine.

#### **Figure S14.** ESI of *N*-(diphenylphosphaneyl)-*N*-1,1-triphenylphosphanamine.


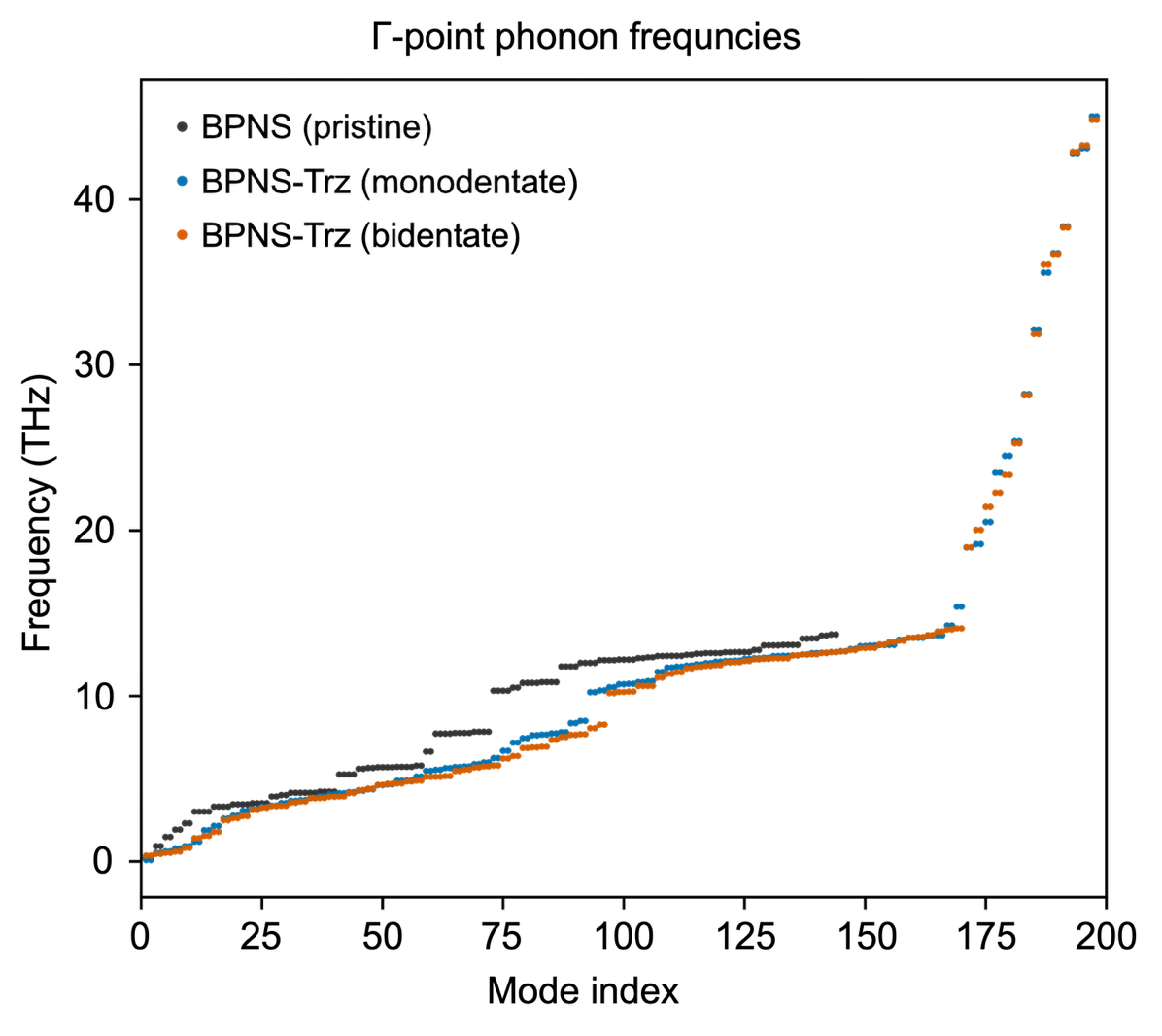


**Figure S15.** Γ-point phonon frequencies of pristine BPNS and mono- and bidentate BPNS-Trz structures calculated using DFPT.

All spin-polarized density functional theory (DFT) calculations were performed using the Vienna ab initio simulation package (VASP).^[8]^ The electronic exchange-correlation energy was treated within the generalized gradient approximation (GGA) using the Perdew-Burke-Ernzerhof (PBE)^[9]^ functional, while long-range interlayer interactions were accounted for through a DFT-D3^[10]^ dispersion correction scheme with Becke-Johnson damping.^[11]^ To implement a frozen-core approximation, core-valence interactions were described using the projector augmented wave (PAW)^[12,13]^ method, where the 3s and 3p electrons of P and Cl, as well as the 2s and 2p electrons of C and N, were treated explicitly as valence electrons. The plane-wave basis was expanded up to a kinetic-energy cutoff of 500 eV. The lattice parameters of pristine black phosphorus were obtained from structural relaxations performed using a Γ-centered K-point mesh of 12 x 12 x 12, with ionic positions optimized until an energy convergence criterion of 10^-5^ eV was achieved. To investigate the site-specific adsorption behavior of 4,6-dichloro-1,3,5-triazine on the surface of black phosphorus, a periodic model was set up with a 3 x 2 supercell of pristine bilayer black phosphorus, comprising 48 P atoms. The Brillouin zone was represented with a Γ-centered K-point sampling of 6 x 6 x 1 while only the top layer of the model was allowed to relax freely in all Cartesian directions. A vacuum spacing of 25 Å was introduced in a direction perpendicular to the bilayer surface to avoid spurious interlayer interactions. The adsorbate molecule with the chemical formula C_3_N_4_Cl_2_ was placed approximately 2.2 Å, as a starting orientation in a perpendicular fashion, above the top layer of the pristine black phosphorus bilayer, with the reactive N site pointed to the surface. Low and high molecular coverages were defined as the adsorption of one and two C_3_N_4_Cl_2_ molecules per 12 available P atoms on the surface. The reaction energies of the modeled process were calculated as:

| ∆E = E(BPNS - nTrz) + nE(N_2_) - E(BPNS) - nE(Trz-N_3_) | (1) |
| --- | --- |
|  |  |

which represents the thermodynamic stability of each isomer (BPNS-Trz) formed on covalent functionalization of a pristine black phosphorus bilayer (BPNS) through reaction with 2-azido-4,6-dichloro-1,3,5-triazine (Trz-N_3_) and a subsequent release of molecular nitrogen (N_2_). This expression is used to compare the relative stability of the resulting BPNS-Trz binding motifs. Therefore, the energetic preference is independent of the inclusion of N₂, as both configurations are evaluated with the same reference. The parameter n indicates the number of molecules of C_3_N_4_Cl_2_ included in the modelling of BPNS-Trz and therefore regulates the low and high adsorbate coverages defined earlier. To perform a qualitative analysis of the binding mode in each BPNS-Trz isomer, atomic charges in the system were determined using a Bader charge analysis scheme,^[14]^ which enables the evaluation of the charge transfer between the bonding P-N sites in the system. To evaluate the energy landscape over the course of the reaction, a potential energy scan (PES) was performed, initiated from Isomer II to Isomer I, with intermediate configurations obtained through unidirectional lateral shifts of the triazine moiety across the BP surface. To probe vibrational eigenmodes and interpret second-order linear response to ionic displacements, phonon calculations were performed using density functional perturbation theory (DFPT)^[15]^ with a tighter energy convergence threshold of 10^-6^ eV and a 3 x 3 x 1 K-mesh.

All calculated phonon frequencies were positive and confirm that the optimized structures correspond to dynamically stable local minima. In Figure S15, the Γ-point phonon frequencies for pristine black phosphorus and for the mono- and bidentate functionalized systems are presented, all calculated using DFPT within a 3 × 2 supercell of pristine black phosphorus. For pristine black phosphorus, the calculated phonon frequencies extended up to approximately 460 cm^-1^ , in agreement with previous reports.^[16]^ On functionalization, a slight reduction in the low frequency modes of phosphorus character was observed, accompanied by the appearance of distinct higher-frequency molecular vibrational modes in the range of 600-1500 cm^-1^.

Infrared-active mode intensities were evaluated by post-processing the DFPT results to extract the Born effective charge tensors and compute the associated oscillator strengths within the dipole approximation.

The optimized structures for the different systems are available from the authors by request.

**
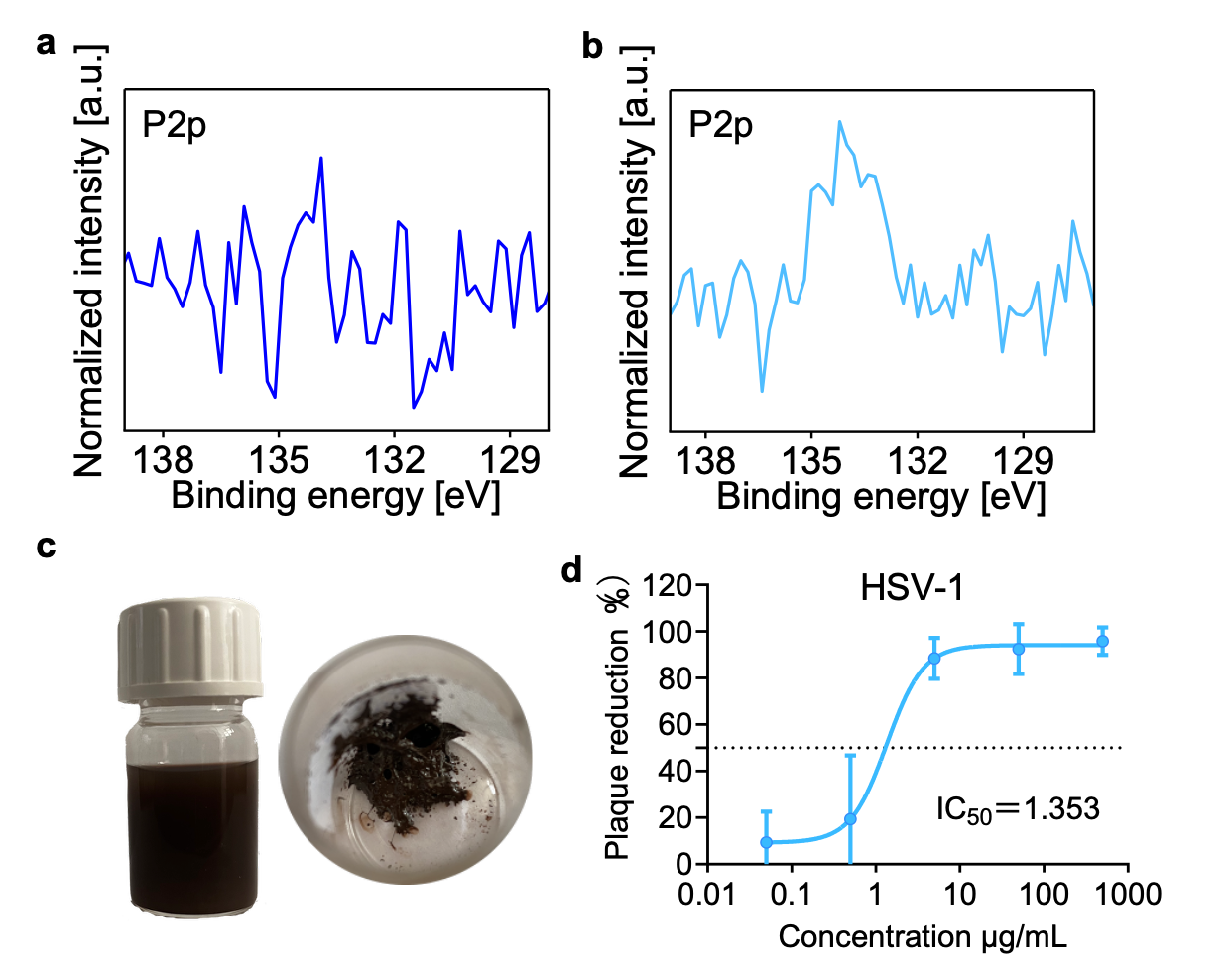
**

**Figure S16.** High resolution XPS P2p spectra of (a) BPNS-Trz-lPG_30_ and (b) BPNS-Trz-lPGS_30_. (c) BPNS-Trz-lPG_7_ in water (1 mg mL^-1^) (left) and lyophilized (right). (d) Results of the plaque reduction assay of BPNS-Trz-lPG_7_ against HSV-1 for IC_50_ determination on Vero E6 cells.


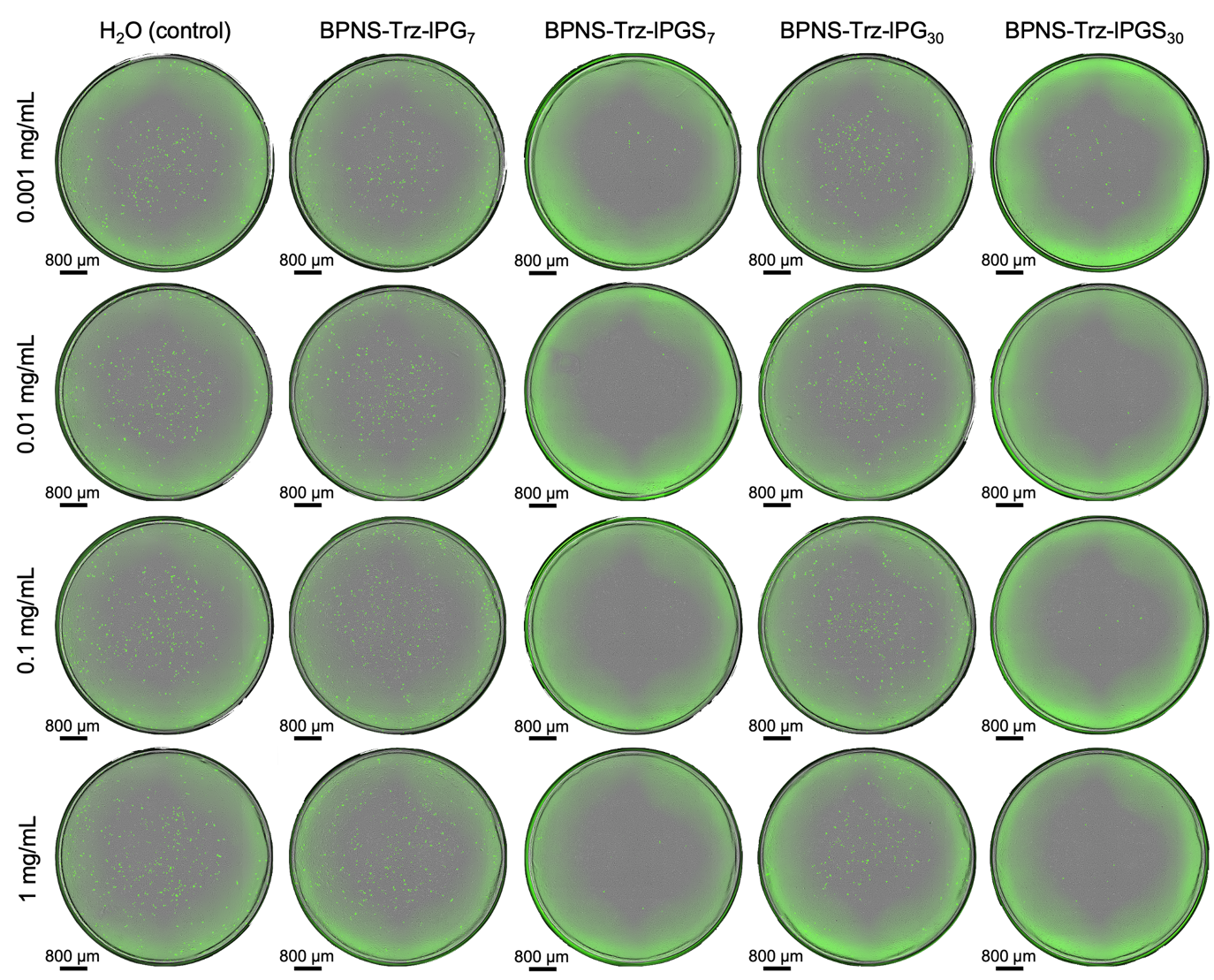


**Figure S17.** Comparison of BP-inhibitors against RSV-GFP infection on A549 cells at different concentrations. RSV-GFP was mixed with compounds at indicated concentrations for 45 min and then incubated with A549 cells for 2 h. Viruses mixed with double-distilled water (ddH_2_O, the solvent of compounds) were used as the control group. The inoculum was then replaced with fresh medium. 24 h later, cells were imaged, and the number of infected cells (with GFP signal) was detected using fluorescence microscopy. scale bar: 800 μm.


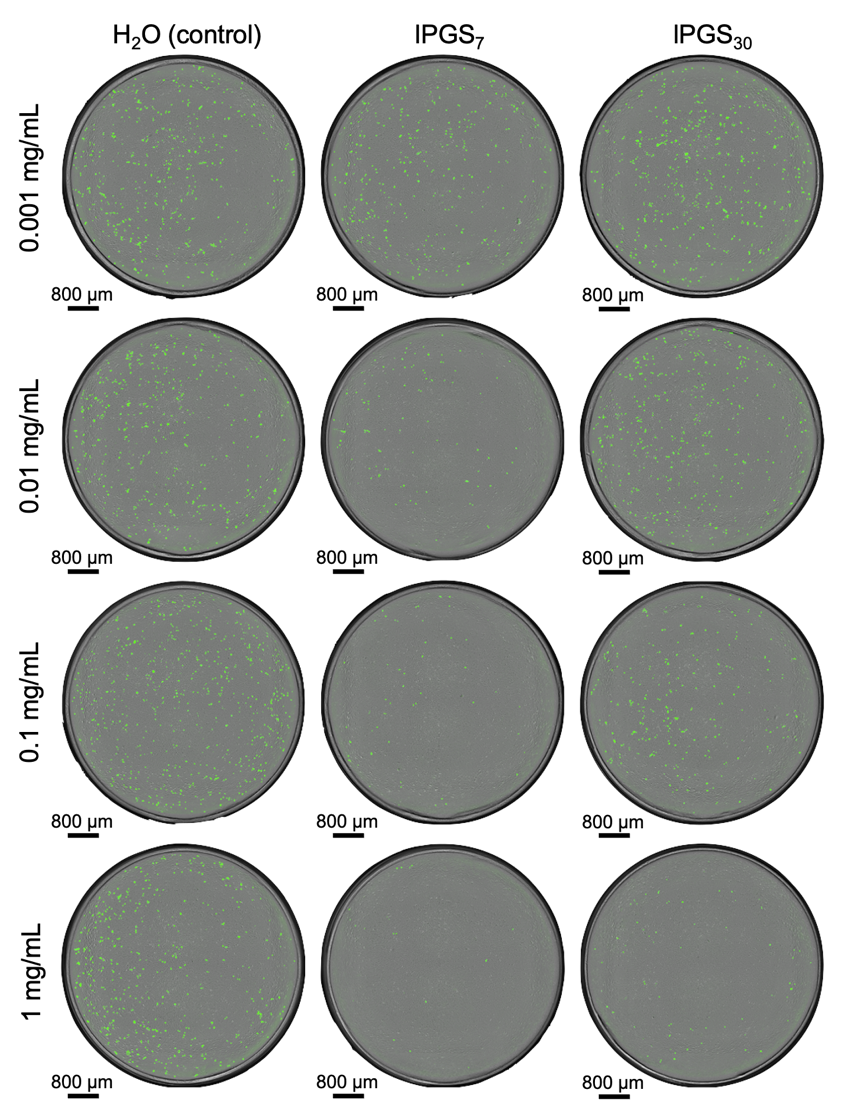


**Figure S18.** Comparison of polymer-inhibitors against RSV-GFP infection on A549 cells at different concentrations. RSV-GFP was mixed with compounds at indicated concentrations for 45 min and then incubated with A549 cells for 2 h. Viruses mixed with double-distilled water (ddH_2_O, the solvent of compounds) were used as the control group. The inoculum was then replaced with fresh medium. 24 h later, cells were imaged, and the number of infected cells (with GFP signal) was detected using fluorescence microscopy. scale bar: 800 μm.

#### **Figure S19.** ^1^H-NMR (500 MHz) spectrum of BP-Trz-lPG_7_ in D_2_O.

**Figure S20.** ^1^H-NMR (500 MHz) spectrum of BP-Trz-lPG_30_ in D_2_O.

#### **Figure S21**. ^1^H-NMR (500 MHz) spectrum of BP-Trz-lPGS_7_ in D_2_O.

**Figure S22.** ^1^H-NMR (500 MHz) spectrum of BP-Trz-lPGS_30_ in D_2_O.

.

#### **Figure S23.** ^13^C-NMR (125 MHz) spectrum of BP-Trz-lPG_7_ in D_2_O.

#### **Figure S24.** ^13^C-NMR (125 MHz) spectrum of BP-Trz-lPG_30_ in D_2_O.

# References

[1] M.-A. Rameix-Welti, R. Le Goffic, P.-L. Hervé, J. Sourimant, A. Rémot, S. Riffault, Q. Yu, M. Galloux, E. Gault, J.-F. Eléouët, “Visualizing the replication of respiratory syncytial virus in cells and in living mice” *Nat Commun* **2014**, *5*, 5104.

[2] A. Sharma, R. Sheyi, A. Kumar, A. El-Faham, B. G. De La Torre, F. Albericio, “Investigating Triorthogonal Chemoselectivity. Effect of Azide Substitution on the Triazine Core” *Org. Lett.* **2019**, *21*, 7888–7892.

[3] T. Meguro, N. Terashima, H. Ito, Y. Koike, I. Kii, S. Yoshida, T. Hosoya, “Staudinger reaction using 2,6-dichlorophenyl azide derivatives for robust aza-ylide formation applicable to bioconjugation in living cells” *Chem. Commun.* **2018**, *54*, 7904–7907.

[4] S. C. Eady, T. Breault, L. Thompson, N. Lehnert, “Highly functionalizable penta-coordinate iron hydrogen production catalysts with low overpotentials” *Dalton Trans.* **2016**, *45*, 1138–1151.

[5] G. Socrates, *Infrared and Raman characteristic group frequencies: tables and charts*, Wiley, Chichester, **2010**.

[6] Z. Guo, H. Zhang, S. Lu, Z. Wang, S. Tang, J. Shao, Z. Sun, H. Xie, H. Wang, X. Yu, P. K. Chu, “From Black Phosphorus to Phosphorene: Basic Solvent Exfoliation, Evolution of Raman Scattering, and Applications to Ultrafast Photonics” *Adv Funct Materials* **2015**, *25*, 6996–7002.

[7] P. Nickl, J. Radnik, W. Azab, I. S. Donskyi, “Surface characterization of covalently functionalized carbon-based nanomaterials using comprehensive XP and NEXAFS spectroscopies” *Applied Surface Science* **2023**, *613*, 155953.

[8] G. Kresse, J. Furthmüller, “Efficient iterative schemes for *ab initio* total-energy calculations using a plane-wave basis set” *Phys. Rev. B* **1996**, *54*, 11169–11186.

[9] J. P. Perdew, K. Burke, M. Ernzerhof, “Generalized Gradient Approximation Made Simple [Phys. Rev. Lett. 77, 3865 (1996)]” *Phys. Rev. Lett.* **1997**, *78*, 1396–1396.

[10] S. Grimme, J. Antony, S. Ehrlich, H. Krieg, “A consistent and accurate *ab initio* parametrization of density functional dispersion correction (DFT-D) for the 94 elements H-Pu” *The Journal of Chemical Physics* **2010**, *132*, 154104.

[11] S. Grimme, S. Ehrlich, L. Goerigk, “Effect of the damping function in dispersion corrected density functional theory” *J Comput Chem* **2011**, *32*, 1456–1465.

[12] G. Kresse, D. Joubert, “From ultrasoft pseudopotentials to the projector augmented-wave method” *Phys. Rev. B* **1999**, *59*, 1758–1775.

[13] P. E. Blöchl, “Projector augmented-wave method” *Phys. Rev. B* **1994**, *50*, 17953–17979.

[14] G. Henkelman, A. Arnaldsson, H. Jónsson, “A fast and robust algorithm for Bader decomposition of charge density” *Computational Materials Science* **2006**, *36*, 354–360.

[15] S. Baroni, S. De Gironcoli, A. Dal Corso, P. Giannozzi, “Phonons and related crystal properties from density-functional perturbation theory” *Rev. Mod. Phys.* **2001**, *73*, 515–562.

[16] Y. Machida, A. Subedi, K. Akiba, A. Miyake, M. Tokunaga, Y. Akahama, K. Izawa, K. Behnia, “Observation of Poiseuille flow of phonons in black phosphorus” *Science Advances* **2018**, *4*, eaat3374.
